# Effects of diffusion MRI spatial resolution on human brain short-range association fiber reconstruction and structural connectivity estimation

**DOI:** 10.1162/IMAG.a.1089

**Published:** 2026-01-14

**Authors:** Jialan Zheng, Ziyu Li, Wen Zhong, Ziang Wang, Zihan Li, Hongjia Yang, Mingxuan Liu, Xiaozhi Cao, Congyu Liao, David H. Salat, Susie Y. Huang, Qiyuan Tian

**Affiliations:** School of Biomedical Engineering, Tsinghua University, Beijing, China; Oxford Centre for Integrative Neuroimaging, FMRIB, Nuffield Department of Clinical Neurosciences, University of Oxford, Oxford, United Kingdom; Department of Radiology, Stanford University, Stanford, CA, United States; Department of Radiology & Biomedical Imaging, University of California San Francisco, San Francisco, CA, United States; Athinoula A. Martinos Center for Biomedical Imaging, Department of Radiology, Massachusetts General Hospital, Charlestown, MA, United States; Harvard Medical School, Boston, MA, United States

**Keywords:** U-fiber, superficial white matter, tractography, sub-millimeter resolution, diffusion tensor imaging, constrained spherical deconvolution

## Abstract

Short-range association fibers (SAFs) are critical for cortical communications but are often underestimated in conventional resolution diffusion magnetic resonance imaging (dMRI) since they locate within a ~1.5 mm thin layer of superficial white matter. With the advent of high-resolution diffusion imaging techniques, this study evaluated the effects of image spatial resolution on SAF reconstruction using two datasets: (1) prospectively acquired dMRI data from 20 healthy subjects, each scanned at 3 resolutions (i.e., 2, 1.5, and 0.96 mm iso.), and (2) retrospectively down-sampled dMRI data from the Human Connectome Project dataset, as well as 20 representative MRtrix3-based tractography pipelines. It was found that lower resolution degraded superficial white matter model fitting, lowered the SAF streamline counts, and reduced global and regional short-range connectivity fraction (SCF), defined as the fraction of SAF connections among all association fiber connections, across all tested methods. Temporal lobe cortical regions exhibited the greatest SCF declines at lower resolutions. Tractography methods differed in resolution sensitivity, with diffusion tensor imaging (DTI)-based single-tissue single-fiber tractography showing greater decreases in SCF than constrained spherical deconvolution (CSD)-based multi-tissue multi-fiber tractography at lower resolutions. Probabilistic, anatomically constrained tractography combined with spherical-deconvolution informed filtering of tractograms was more robust to decreases in resolution. Up-sampling to a nominally higher resolution partially improved model fitting and SCF accuracy across the evaluated pipelines, with the greatest effect observed for DTI. Using the 0.96 mm iso. gSlider data and optimized tractography pipelines from this study, we constructed the first human brain atlas of RSCF. In summary, this study provides a systematic and quantitative evaluation using MRtrix3 of how spatial resolution, fiber models, and tracking methodologies affect SAF reconstruction and structural connectivity estimation, serving as a reference framework for methodological choices. These advances may enhance the characterization of both healthy and diseased human brains across a wide range of neuroscientific and clinical applications.

## 1 Introduction

Short-range association fibers (SAFs) are white matter (WM) pathways that connect adjacent gyri within the same hemisphere (Guevara et al., 2020). These fibers typically span between 3 and 30 mm in length and are primarily located within a ~1.5 mm thin layer of superficial WM beneath the cerebral cortex (Kirilina et al., 2020; Movahedian Attar et al., 2020; Shastin et al., 2022). Due to their characteristic shape following cortical gyrification, SAFs are commonly referred to as U-fibers (Tian et al., 2025; Van Dyken et al., 2024).

SAFs are important for understanding the cognitive function and developmental and neurodegenerative mechanisms of the human brain. First, they occupy ~240 cm^3^ of the total ~420 cm^3^ WM volume and constitute 90% of the brain’s axonal fibers (Schüz & Miller, 2002), rendering them essential for cortico-cortical communications (Markov et al., 2013). Additionally, due to their latest myelination pattern (Barkovich, 2000; Parazzini et al., 2002; M. Wu et al., 2016) and the “last-in-first-out” principle of neurodegeneration (Braak & Braak, 1996; Fjell et al., 2014), SAFs are among the first structures to show pathological changes in neurodegenerative diseases such as Alzheimer’s (Carmeli et al., 2014; Fornari et al., 2012).

Fiber tracking (tractography) based on diffusion MRI (dMRI) is the most widely used method for *in vivo* mapping of SAFs. DMRI measures the diffusion patterns of water molecules in brain tissue (Le Bihan et al., 1986), which are utilized to infer local microstructure properties (Assaf et al., 2008; Le Bihan et al., 2001; H. Zhang et al., 2012). Tractography integrates local information across voxels to reconstruct continuous fiber pathways and whole-brain connectomes (Mori & Van Zijl, 2002). Pioneering tractography approaches (Mori et al., 1999) relied on principal diffusion direction derived from diffusion tensor imaging (DTI), which are intrinsically limited in resolving crossing fibers. Therefore, more sophisticated methods were developed to model crossing fiber configurations. For instance, the ball-and-stick model separates diffusion signal into isotropic (ball) and anisotropic (stick) components (Behrens et al., 2007; Jbabdi et al., 2012). Similarly, constrained spherical deconvolution (CSD) models each fiber population using an empirically determined response function, or even multiple response functions for different tissue types (Jeurissen et al., 2014; Tournier et al., 2007).

The accuracy of SAF reconstruction using tractography is hampered by the limited spatial resolution of dMRI. The most widely adopted 2D EPI-based dMRI protocol usually employs 2–3 mm iso. spatial resolution and a single b-value of 800–1500 s/mm^2^ for DTI (W. Wu & Miller, 2017) due to the inherent trade-off between the macro- and micro-anatomy resolving capability and signal-to-noise ratio (SNR). These spatial and diffusion resolutions are insufficient for capturing the microstructural patterns of SAFs residing in the ~1.5 mm thin layer of superficial WM (González Ballester et al., 2002), which are even thinner than most cortices. Mixing with gray matter (GM) signals may cause premature termination of tractography or increased uncertainty in probabilistic tracking algorithms, while mixing with deep WM signals may cause SAF tracking to erroneously extend into deeper regions (Reveley et al., 2015), both leading to underestimated SAF connectivity. To map SAFs accurately, high spatial resolution (1 mm iso. or higher) is expected to reduce partial volume effects (Movahedian Attar et al., 2020; Song et al., 2014), whereas high diffusion-encoding sensitivity (2000 s/mm^2^ or higher) tends to aid tractography in resolving fiber crossings (Behrens et al., 2007; Dell’Acqua & Tournier, 2019; Jbabdi et al., 2012).

Recent advances in dMRI acquisition have significantly improved imaging resolution. 3D EPI enhances SNR efficiency by shortening TR and enables high iso. resolution with 3D Fourier encoding, which was first demonstrated useful in *ex vivo* imaging at 0.7 mm iso. resolution (K. L. Miller et al., 2011). For *in vivo* imaging with unavoidable subject motion, 3D multi-slab EPI was developed to mitigate motion artifacts. Each excited slab is corrected for motion-induced shot-to-shot phase variation with a 2D navigator (Engström & Skare, 2013; W. Wu et al., 2016). Recently, this technique was further developed to enable 0.53 mm iso. resolution for *in vivo* dMRI (Z. Li et al., 2026). Another representative technique Generalized Slice Dithered Enhanced Resolution (gSlider) acquires multiple thick slabs using various RF excitations while maintaining high in-plane resolution. These RF-encoded thick slices are then used to compute high-resolution thin slices as an inverse problem with Tikhonov regularization. GSlider improved SNR through multiple excitations and achieved 0.76 mm iso. resolution for *in vivo* dMRI (Setsompop et al., 2018). More recently, Romer-EPTI enabled 0.5 mm iso. resolution for *in vivo* dMRI through super-resolution reconstruction of multiple thick-slice volumes with rotated field of view (Dong et al., 2025).

Although prior high-resolution dMRI studies have reported accurate SAF reconstruction (Reveley et al., 2015; Song et al., 2014; F. Zhang et al., 2024), systematic, quantitative, and direct evaluations across spatial resolutions and tractography pipelines remain limited. Many current SAF studies have nevertheless used conventional low-resolution protocols (K. G. Schilling et al., 2023; Urquia-Osorio et al., 2022; Yuan et al., 2022). The impact of up-sampling on SAF reconstruction is also unclear: while some studies suggested that up-sampling dMRI data prior to modeling and analysis benefit DTI-based tractography (Dyrby et al., 2014), others indicated that data up-sampling may yield results inferior to natively acquired high-resolution data for CSD-based tractography (Dmitri et al., 2019). To bridge these gaps, we quantitatively evaluated the impact of dMRI spatial resolution on reconstructing SAFs and estimating structural connectivity, using within-subject, multi-resolution data across 20 commonly adopted tractography pipelines. Our hypothesis is that higher resolution benefits SAF reconstruction through reduced partial volume effects.

Specifically, we comprehensively compared voxel-wise model fitting results, whole-brain tractography results, and structural connectivity estimation results across multiple commonly adopted spatial resolutions. We focused on MRtrix-based pipelines (Tournier et al., 2019), which were widely used in brain developmental studies (Grotheer et al., 2022), atlas construction (Radwan et al., 2022), evolutionary neuroscience (Gerussi et al., 2023), surgical target localization (Lehman et al., 2020), and clinical prognostic analyses (Pruckner et al., 2023). We analyzed two datasets: (i) prospectively acquired dMRI data at 1.5 and 2 mm iso. resolution using the vendor-provided product 2D EPI sequence, and at 0.96 mm iso. resolution using the gSlider sequence, and (ii) retrospectively down-sampled dMRI data from 1.25 mm to 1.5 and 2 mm iso. resolution provided by the Human Connectome Project (HCP) WU-Minn consortium (Van Essen et al., 2013). We also assessed how fiber models (single or crossing) and tracking methodologies (deterministic or probabilistic, with or without spherical-deconvolutional informed filtering, with or without anatomy constraints) influence across-resolution differences and their cortical heterogeneity. These findings clarify how dMRI spatial resolution affects short-range and whole-brain structural connectivity estimation, inform dMRI acquisition and analysis choices, and help advance understanding of SAFs, human brain networks, and their alterations.

## 2 Material and Methods

### 2.1 Prospectively acquired data

With approval from the Tsinghua University Institutional Review Board and written informed consent, MRI data were acquired from 20 healthy young adults (mean age: 23.45 ± 1.80 years; 12 females) on a Siemens MAGNETOM Prisma 3T scanner equipped with a 32-channel head coil. T_1_-weighted (T1w) MPRAGE (1 mm iso.) and dMRI data at multiple iso. resolutions (0.96, 1.5, and 2 mm iso.) were acquired for each subject.

The sub-millimeter resolution (0.96 mm iso.) diffusion data were acquired using the gSlider sequence (Setsompop et al., 2018). This sequence acquired five 4.8 mm-thick slabs with 0.96 × 0.96 mm^2^ in-plane resolution using five optimized and varying RF profiles for excitation, which were then used to reconstruct 0.96 mm-thick slices for achieving 0.96 mm iso. spatial resolution. To minimize slab-boundary artifacts, low-resolution B1+ maps were acquired to calibrate the RF encoding profiles to account for incomplete T_1_ recovery effects due to B1+ field inhomogeneity (Liao et al., 2020). The diffusion protocol included 32 diffusion-weighted images (DWIs) at b = 1000 s/mm^2^ and 64 DWIs at b = 2500 s/mm^2^ along uniformly distributed diffusion directions. A b = 0 image was inserted after every 16 DWIs. An additional b = 0 image with reversed phase-encoding direction was acquired for susceptibility distortion correction.

Diffusion data at commonly adopted 1.5 and 2 mm iso. resolution were acquired using the product 2D simultaneous multi-slice (SMS) pulsed gradient spin echo (PGSE) single-shot EPI sequence (i.e., ep2d sequence). The diffusion-encoding scheme (b-values and directions) and phase-encoding strategy were identical to those used in the gSlider sequence. Detailed acquisition parameters are provided in Table 1.

**Table 1.** Acquisition parameters for prospectively acquired data.

	T1w MP-RAGE	gSlider	ep2d_1p5	ep2d_2
Repetition/Echo Time (ms)	2530 / 2.27	3900 / 80	5400 / 76	5400 / 76
Field of View (mm^2^)	256 × 256	200 × 200	200 × 200	200 × 200
In-Plane Resolution (mm^2^)	1 × 1	0.96 × 0.96	1.5 × 1.5	2 × 2
Slice Thickness (mm)	1	0.96	1.5	2
Diffusion Encoding	–	10 × b = 032 directions × b = 1000 s/mm^2^ 64 directions × b = 2500 s/mm^2^		
In-plane Accel. Factor (GRAPPA R)	2	3	2	2
Acquisition Time (min:sec)	6:03	35:06	10:51	10:33

All diffusion MRI data were preprocessed for distortion correction and image alignment. First, gradient nonlinearity correction was applied (Janke et al., 2004). Then, the susceptibility-induced off-resonance field was estimated using b = 0 images acquired with opposite phase encoding directions by the “topup” function from the FMRIB Software Library (FSL) (Andersson et al., 2003; Graham et al., 2017). This field map, along with the diffusion data, was input to FSL’s “eddy” function for correcting susceptibility and eddy current-induced distortions and subject motion (Andersson et al., 2016). Finally, to investigate the effects of up-sampling (Dyrby et al., 2014), the preprocessed data at 1.5 and 2 mm iso. resolution were up-sampled to 0.96 mm iso. using FSL’s “flirt” function with spline interpolation.

T1w images were also corrected for gradient nonlinearity. Cortical surface reconstruction and volumetric segmentation were performed using FreeSurfer’s “recon-all” function (Fischl, 2012). The preprocessed diffusion data and T1w data were co-registered using the boundary-based registration implemented in FreeSurfer’s “bbregister” function (Greve & Fischl, 2009).

### 2.2 Retrospectively down-sampled data

To complement the analysis of prospectively acquired data, pre-processed dMRI data (1.25 mm iso.) from 20 healthy HCP subjects (Van Essen et al., 2013) were retrospectively down-sampled to 1.5 and 2 mm iso. resolution and analyzed. Co-registered T1w data (0.7 mm iso.) were also used to assist tractography. The dMRI protocol included 18 b = 0 volumes and 90 DWIs for each of the 3 shells (b = 1000, 2000, 3000 s/mm²), with acquisition and preprocessing details previously described (Glasser et al., 2013; Sotiropoulos et al., 2013; Uğurbil et al., 2013).

Down-sampling was performed in the Fourier domain of the 3D images by truncating high spatial-frequency components to the target matrix size, followed by mild apodization to reduce Gibbs ringing artifacts. The resulting low-resolution images were then up-sampled to 1.25 mm iso. resolution using the same procedure applied to the prospectively acquired data.

### 2.3 Connectivity quantification

**Fig. 1.**
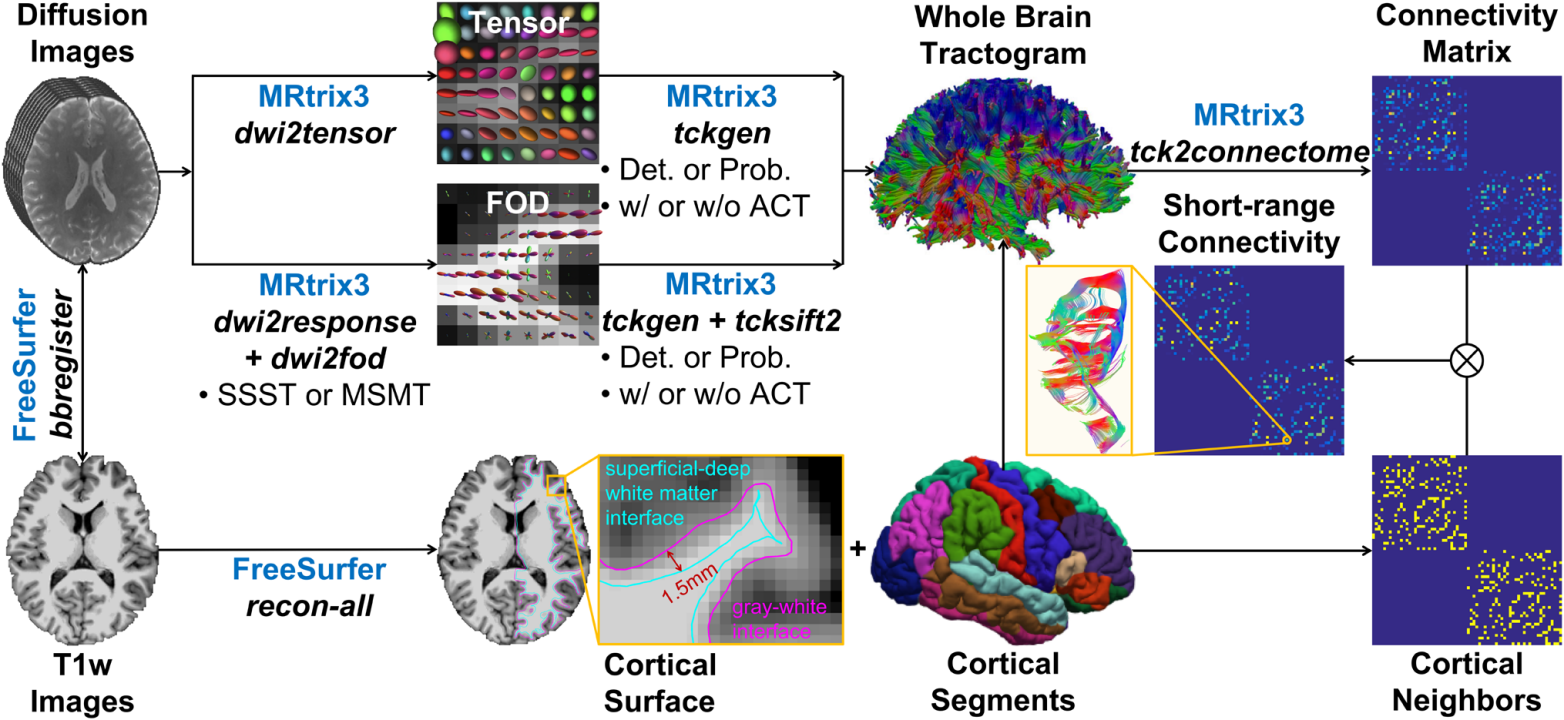
Structural connectivity estimation overview. Tractography was performed using the MRtrix3 software on single-shell or multi-shell prospectively acquired and retrospectively down-sampled diffusion data using (1) single-fiber (tensor modeling) or crossing-fiber modeling (single-shell single-tissue (SSST) or multi-shell multi-tissue (MSMT) constrained spherical deconvolution (CSD)) and (2) deterministic or probabilistic tracking, (3) with or without spherical-deconvolution informed filtering of tractograms (SIFT), (4) with or without anatomically constrained tractography (ACT) for estimating structural connectivity assisted by the FreeSurfer software.

Multiple tractography pipelines from the MRtrix3 software were adopted for estimating SAF pathways and structural connectivity (Fig. 1). Specifically, tractography was performed on single-shell or multi-shell data using (1) single-fiber (tensor) or crossing-fiber (constrained spherical deconvolution (CSD)) modeling, (2) deterministic or probabilistic tracking, (3) with or without spherical-deconvolution informed filtering of tractograms (SIFT), and (4) with or without anatomically constrained tractography (ACT). SIFT and ACT enhance the biological plausibility of reconstructed fiber pathways by assigning per-streamline weights, making the weighted streamline density along each fiber direction proportional to the corresponding fiber orientation distribution (FOD) amplitude (Smith et al., 2013, 2015) and forcing them to initiate and terminate at the interface of WM and cortical or deep GM, with seeds distributed uniformly across the GM–WM interface rather than uniformly throughout the brain volume (Smith et al., 2012), respectively. In total, 20 tractography strategies were used (Table 2). Tractography parameters were selected using the software’s default settings (Tournier et al., 2019) on the highest-resolution data and were then kept identical across all spatial resolutions (Supplementary Table S1 for prospectively acquired data and Supplementary Table S2 for retrospectively down-sampled data). For the single-shell data, data with b = 1000 s/mm^2^ or b = 2500 s/mm^2^ from prospectively acquired data and data with b = 1000 s/mm^2^ or b = 2000 s/mm^2^ from retrospectively down-sampled data were used. For the multi-shell data, data with b = 1000 s/mm^2^ and b = 2500 s/mm^2^ from prospectively acquired data and data with b = 1000 s/mm^2^, b = 2000 s/mm^2^ and b = 3000 s/mm^2^ from retrospectively down-sampled data were used.

**Table 2.** Overview of tractography strategies.

	Single-shell data			Multi-shell data	
	Single-fiber model w/o SIFT	Crossing-fiber model w/o SIFT	Crossing-fiber model w/ SIFT	Crossing-fiber model w/o SIFT	Crossing-fiber mode w/ SIFT
**Det.**	Low B, Sing, Det, w/o SIFT, w/o ACT	High B, Xing, Det, w/o SIFT, w/o ACT	High B, Xing, Det, w/ SIFT, w/o ACT	Multi B, Xing, Det, w/o SIFT, w/o ACT	Multi B, Xing, Det, w/ SIFT, w/o ACT
	Low B, Sing, Det, w/o SIFT, w/ ACT	High B, Xing, Det, w/o SIFT, w/ ACT	High B, Xing, Det, w/ SIFT, w/ ACT	Multi B, Xing, Det, w/o SIFT, w/ ACT	Multi B, Xing, Det w/ SIFT, w/ ACT
**Prob.**	Low B, Sing, Prob, w/o SIFT, w/o ACT	High B, Xing, Prob, w/o SIFT, w/o ACT	High B, Xing, Prob, w/ SIFT, w/o ACT	Multi B, Xing, Prob, w/o SIFT, w/o ACT	Multi B, Xing, Prob, w/ SIFT, w/o ACT
	Low B, Sing, Prob, w/o SIFT, w/ ACT	High B, Xing, Prob, w/o SIFT, w/ ACT	High B, Xing, Prob, w/ SIFT, w/ ACT	Multi B, Xing, Prob, w/o SIFT, w/ ACT	Multi B, Xing, Prob, w/ SIFT, w/ ACT

Low B = single-shell with low b-value; High B = single-shell with high b-value; Multi B = multiple shells with both low and high b-values; Sing = single-fiber model; Xing = crossing-fiber model; Det. = deterministic; Prob. = probabilistic; SIFT = spherical-deconvolution informed filtering of tractograms; ACT = anatomically constrained tractography.

The tensor-based tractography was performed on single-shell data with a low b-value (b = 1000 s/mm^2^) using the “tckgen” function from the MRtrix3 software (Tournier et al., 2019) using both deterministic (“Tensor_Det” option) and probabilistic (“Tensor_Prob” option) tracking, without SIFT, and with or without ACT.

The CSD was performed with “dwi2response” and “dwi2fod” functions from the MRtrix3 software (Jeurissen et al., 2014; Tournier et al., 2004, 2007) using single-shell single-tissue (SSST) or multi-shell multi-tissue (MSMT) approaches. SSST-CSD utilized single-shell data with a high b-value (b = 2500 s/mm^2^ for prospectively acquired data and b = 2000 s/mm^2^ for retrospectively down-sampled data) and MSMT-CSD utilized multi-shell data. Subsequently, CSD-based tractography was performed using the “tckgen” function using both deterministic (“SD_Stream” option) and probabilistic (“iFOD2” option) tracking, with or without SIFT (applied with “tcksift2” function in MRtrix3 software) and with or without ACT.

Structural connectivity matrix W was generated using the 68 cortical regions defined by the Desikan–Killiany (DK) atlas (Desikan et al., 2006). The element $\omega_{i,j}$ of the matrix W, representing the connection strength between region i and region j, was defined as

$$\omega_{i,j}=\{\begin{array}{cc} \text{NOS}\left(i,j\right) & \text{if same hemisphere and }i\neq j\\ 0 & \text{otherwise} \end{array},$$ (1)

where NOS(i,j) is the number of streamlines connecting region i and region j. W includes structural connectivity of association fibers but not projection and commissural fibers.

### 2.4 Neighboring cortices identification

To identify short-range connectivity, the neighboring cortices of each cortical region were identified. The neighboring pattern was represented by a matrix A(i,j), with A(i,j)=1 if a cortical region i from the DK atlas borders on cortical region j; otherwise, A(i,j)=0 . Therefore, the connectivity strength $\omega_{i,j}$ at location with A(i,j)=1 in the connectivity matrix W results from SAFs, while $\omega_{i,j}$ at location with A(i,j)=0 results from long-range association fibers.

### 2.5 Connectivity metrics

A quantitative metric termed Short-range Connectivity Fraction (SCF) was adopted to quantify the fraction of SAFs in the connectivity matrix W, following prior work (Ouyang et al., 2016). Since SCF was normalized by the total connectivity strength to mitigate global inflation in streamline counts caused by up-sampling-induced smoothing (Sommer et al., 2017), systematic within-subject differences in SCF across imaging protocols could be interpreted as biases in SAF reconstruction. SCF was calculated at both global and regional levels: global SCF (GSCF) was defined as the ratio of the short-range connectivity strength to the entire connectivity strength:

$$\text{GSCF}=\frac{\sum_{i,j\text{ s.t. }A\left(i,j\right)=\text{1}}\omega_{i,j}}{\sum_{i,j}\omega_{i,j}}$$ (2)

for a particular cortical region k, the regional SCF ${\text{RSCF}}_{k}$ was calculated as the ratio of its short-range connectivity strength to the entire connectivity strength originating from this region:

$${\text{RSCF}}_{k}=\frac{\sum_{j\text{ s.t. }A\left(k,j\right)=\text{1}}\omega_{k,j}}{\sum_{j}\omega_{k,j}}$$ (3)

### 2.6 Statistical analysis

Statistical analysis was performed separately for GSCF and RSCF using Python (scipy 1.13.1, statsmodels 0.14.2). For each tractography method, GSCF was compared across spatial resolutions within subjects. Normality of subject-wise GSCF differences for each resolution pair was assessed using the Shapiro–Wilk test (threshold 0.05). If normal, two-sided paired t-tests were used; otherwise, two-sided Wilcoxon signed-rank tests were used. Within each method, p-values from all cross-resolution comparisons were corrected for multiple testing using the Benjamini–Hochberg false discovery rate (FDR), with significance defined as FDR-corrected p < 0.05. For RSCF, per-region values between 0.96 mm (prospectively acquired data) or 1.25 mm (retrospectively down-sampled data) and 2 mm were compared for each tractography method. Statistical testing followed the same procedure as for GSCF, with p-values across regions corrected using the Benjamini–Hochberg FDR (significance threshold: FDR-corrected p < 0.05).

## 3 Results

**Fig. 2.**
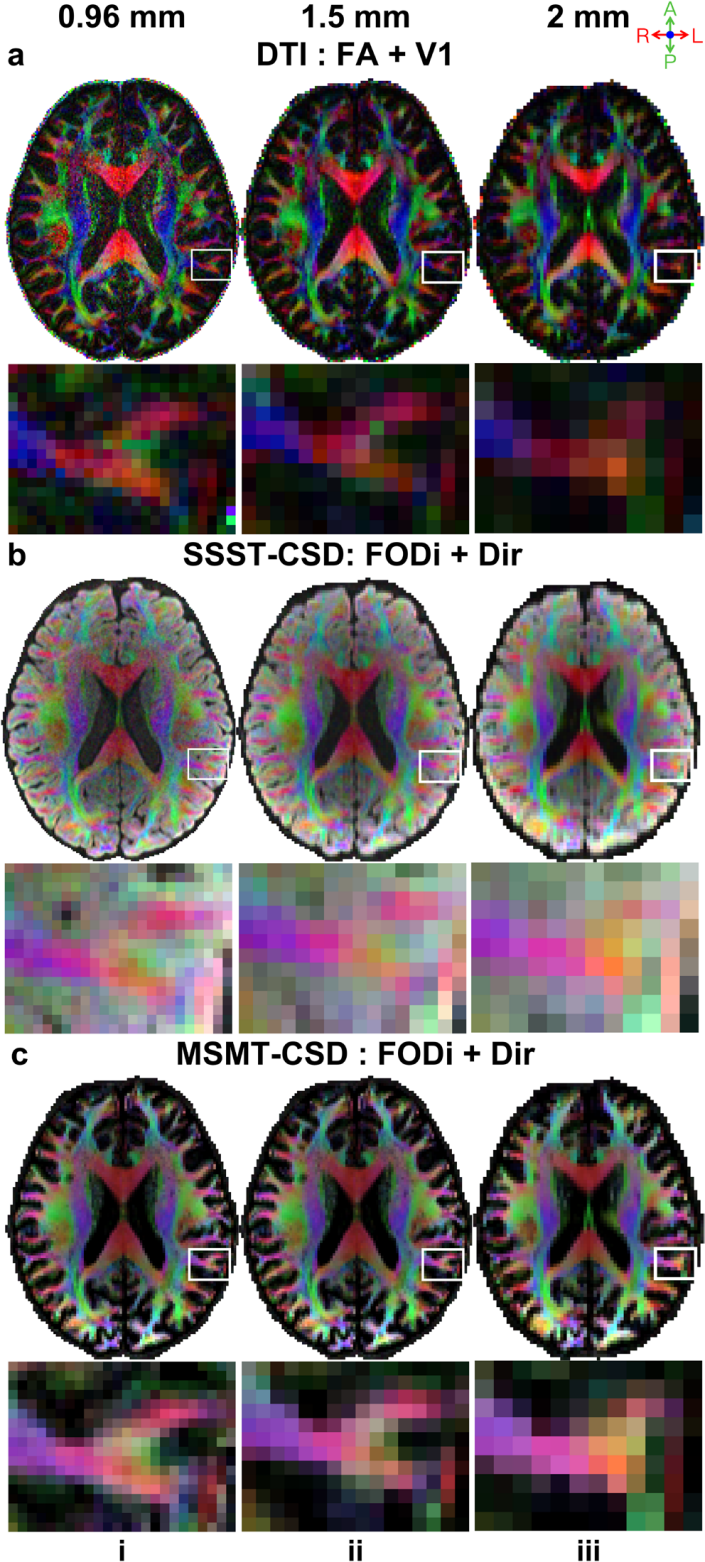
Prospectively acquired data quality. Axial slices from a representative subject with fitted results from three diffusion models (a: DTI, b: SSST-CSD, c: MSMT-CSD) across three resolutions (i: 0.96 mm, ii: 1.5 mm, iii: 2 mm) are displayed. The fractional anisotropy (FA) map color encoded by primary vector (V1) (a) and the fiber orientation distribution integral (FODi) map color encoded by the overall fiber direction (b, c) are shown for DTI and CSD methods (red: left-right; green: anterior-posterior; blue: superior-inferior), respectively.

The prospectively acquired and retrospectively down-sampled data at different spatial resolutions and derived microstructural metrics exhibited high quality, with no noticeable artifacts (Fig. 2; Supplementary Fig. S1). Structural details (e.g., GM-WM interface) were clearer at higher resolution. Temporal signal-to-noise ratio (SNR), defined as the ratio of the mean to the standard deviation of the signal across multiple b = 0 images and averaged within the brain mask (Manzano Patron et al., 2024), decreased with increasing spatial resolution in the present data (2 mm: 40.49; 1.5 mm: 22.21; 0.96 mm: 11.41; values for other data are provided in Supplementary Fig. S5). Nevertheless, SNR was adequate at all resolutions (i.e., SNR ≥ 10) (Manzano Patron et al., 2024). Retrospectively down-sampled data showed the same qualitative trends in image quality, SNR, and derived metrics across spatial resolutions (Supplementary Fig. S1).

**Fig. 3.**
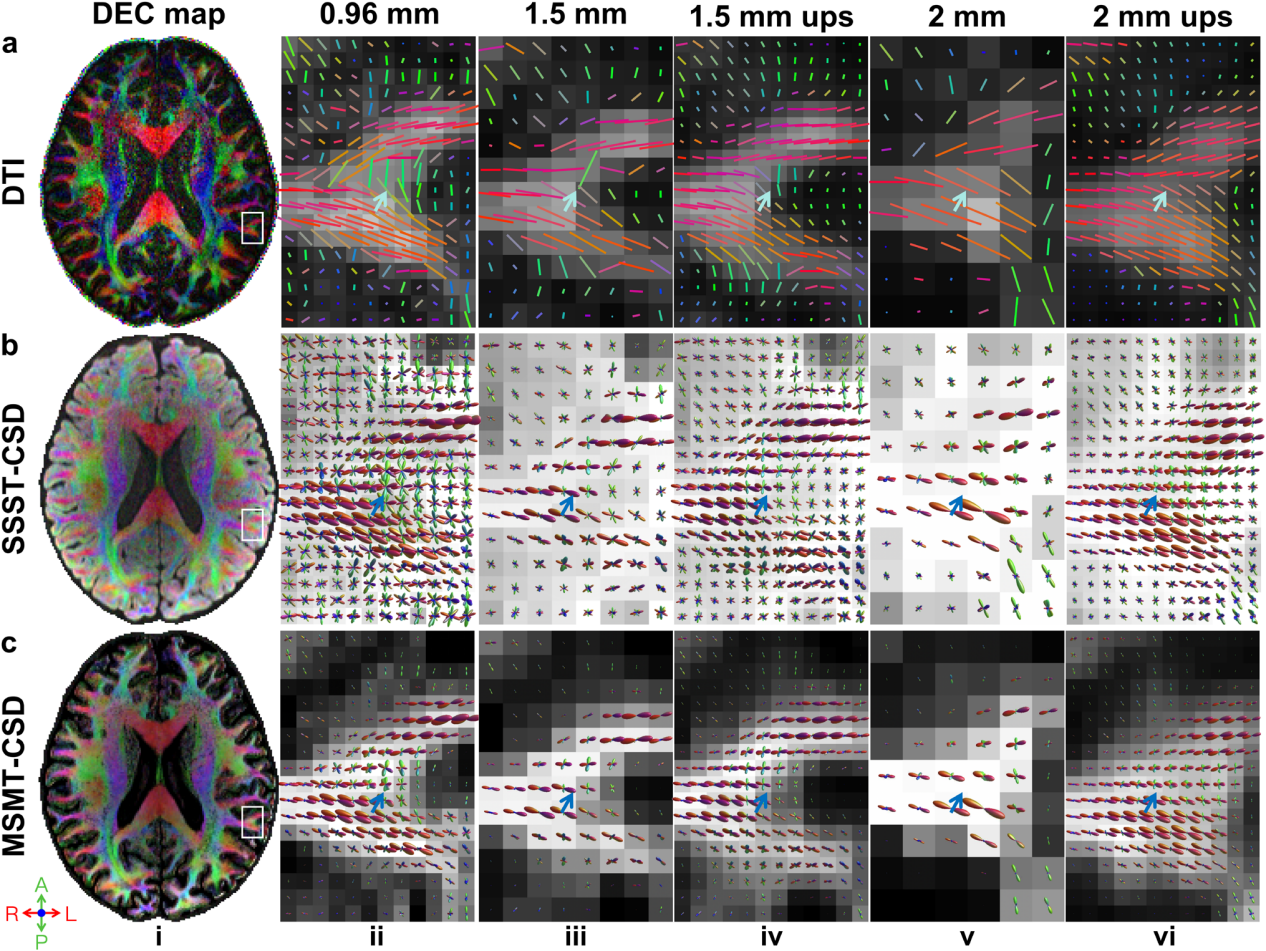
Fiber orientations from prospectively acquired data. Primary fiber direction encoded color (DEC) maps (red: left-right; green: anterior-posterior; blue: superior-inferior) at 0.96 mm iso. resolution from a representative subject (i) are displayed for three methods including DTI (a, i), SSST-CSD (b, i), and MSMT-CSD (c, i), with a region of interest (white boxes) containing gyri, subcortical white matter, and their interface shown in enlarged views overlaid on FA (a), and FOD integral (b, c) maps across three different native spatial resolutions (ii: 0.96 mm, iii: 1.5 mm, v: 2 mm) and two nominally high 0.96 mm iso. resolution up-sampled from 1.5 mm and 2 mm iso. resolutions (iv: 1.5 mm up-sampled, vi: 2 mm up-sampled). Blue arrows highlight a region with short-range association fibers.

Higher spatial resolution substantially improved the representation of superficial WM architecture (Fig. 3; Supplementary Fig. S2). At 0.96 mm iso. resolution, SAFs were clearly delineated by the DTI primary eigenvectors (i.e., primary fiber orientations) (Fig. 3a, ii, blue arrows, green fibers). They diminished as the spatial resolution decreased (Fig. 3a, ii to iii to v) and entirely disappeared at 2 mm iso. resolution (Fig. 3a, v), in which case the superficial WM layer was visually dominated by reddish long-range fibers entering the cortex. Up-sampling the 1.5 mm iso. data increased the number of voxels representing SAFs and thus slightly improved the SAF delineation (Fig. 3a, iii to iv). Up-sampling the 2 mm iso. diffusion data did not recover any SAFs that were originally missing at the native resolution (Fig. 3a, v to vi). The mean FA within the superficial WM layer, quantified using a mask defined at the GM-WM interface (Supplementary Fig. S6), was highest at 0.96 mm iso. resolution (0.37; Fig. 3a, ii) and decreased at lower resolutions (0.36 at 1.5 mm, 0.36 at 1.5 mm up-sampled to 0.96 mm, 0.21 at 2 mm, 0.33 at 2 mm up-sampled to 0.96 mm; Fig. 3a, ii–vi), reflecting partial volume effects with GM and radial long-range fibers entering the cortex.

SAFs were robustly delineated across spatial resolutions by SSST-CSD and MSMT-CSD (Fig. 3b, c), crossing-fiber models that are capable of disentangling SAFs from long-range fibers entering the cortex. However, the FOD magnitude (reflecting the volume fraction) of SAFs gradually reduced as the resolution decreased due to the partial volume effect, potentially hampering the accurate tracking of SAFs and the faithful estimation of short-range connectivity. It is worth noting that the commonly adopted SIFT method filters track density according to the FOD magnitude. Up-sampling the lower resolution data did not recover the lost FOD magnitude of SAFs (Fig. 3b, c, iii to iv and v to vi). Comparing with SSST-CSD results, MSMT-CSD results included much fewer fibers with lower FOD magnitude in the superficial WM (i.e., mainly SAFs) and cortex (Fig. 3c, blur arrows), which were potentially spurious. Moreover, the FOD integral map from MSMT-CSD exhibited clear GM-WM boundaries (Fig. 3b), indicating this model’s capability to distinguish between GM and WM, which were not displayed in SSST-CSD maps. Similar voxel-wise fiber patterns for DTI, SSST-CSD, and MSMT-CSD were observed in retrospectively down-sampled data (Supplementary Fig. S2).

**Fig. 4.**
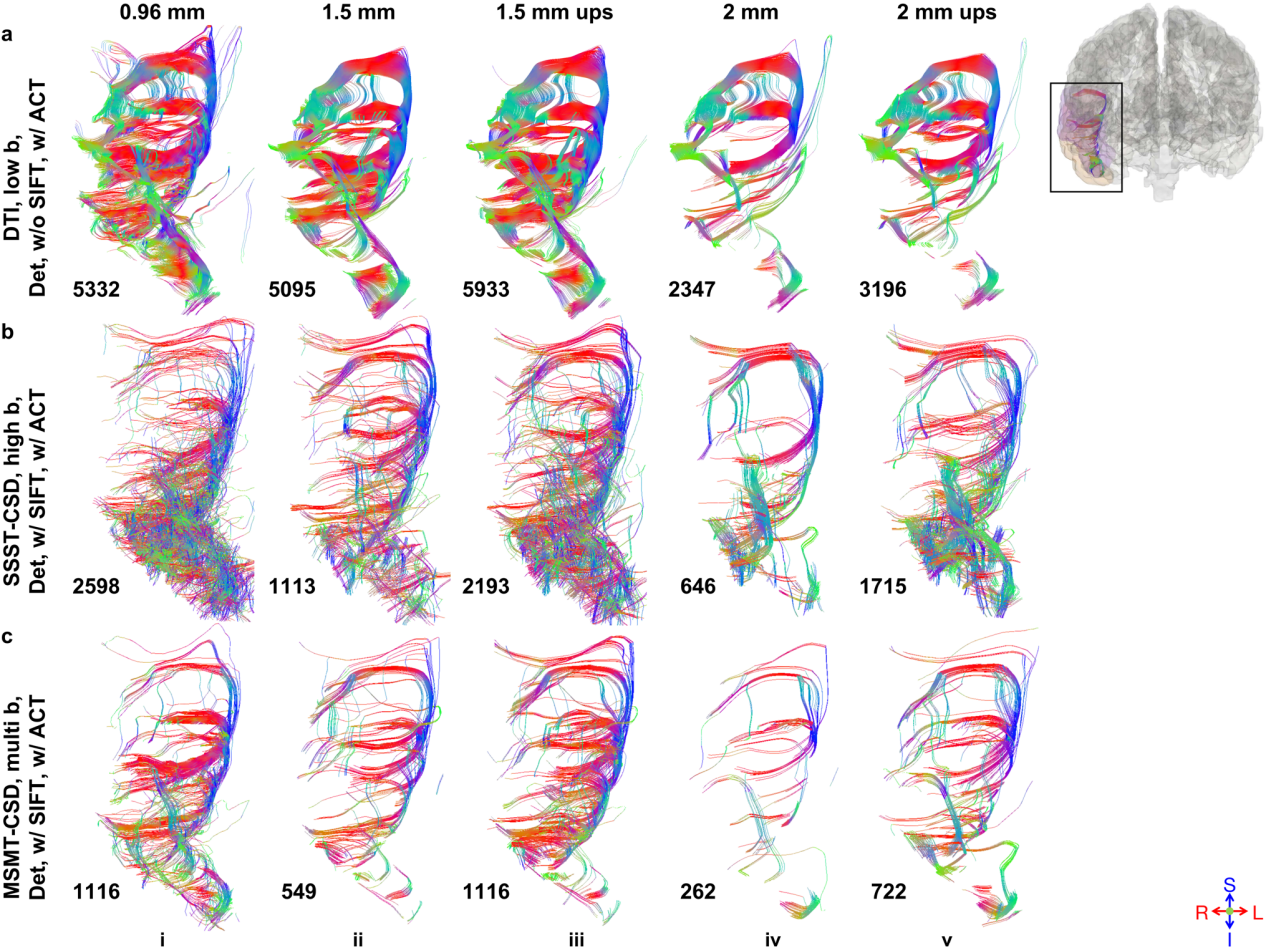
Prospectively acquired data tractography results. U-shaped SAFs connecting the right middle and superior temporal gyri (black box), extracted from whole-brain tractograms reconstructed using the same number of seeds and three modeling methods (a: DTI on low b-value single-shell data, b: SSST-CSD on high b-value single-shell data with SIFT, c: MSMT-CSD on multi b-value data with SIFT), as well as deterministic, anatomically-constrained tractography (ACT) across three different native spatial resolutions (i: 0.96 mm, ii: 1.5 mm, iv: 2 mm) and two nominally high 0.96 mm iso. resolution up-sampled from 1.5 mm and 2 mm iso. resolutions (iii: 1.5 mm up-sampled, v: 2 mm up-sampled) are displayed. Each tract is annotated (lower-left) with either the number of streamlines (a) or the sum of streamline weights (b and c).

U-shaped SAFs were reliably reconstructed by different tractography methods, as confirmed by visual inspection, for example, SAFs connecting the right middle and superior temporal regions (Fig. 4; Supplementary Fig. S3). Streamline counts for each tract decreased with lower spatial resolution and increased after up-sampling (Fig. 4ii to iii and iv to v). The other tractography pipelines exhibited the same resolution-dependent pattern. Among the three exemplar tractography pipelines, DTI produced the most continuous and coherent U-shaped arch (Fig. 4a). MSMT-CSD was relatively coherent (Fig. 4c), whereas SSST-CSD potentially included spurious short fibers at the base of the tract (Fig. 4b).

**Fig. 5.**
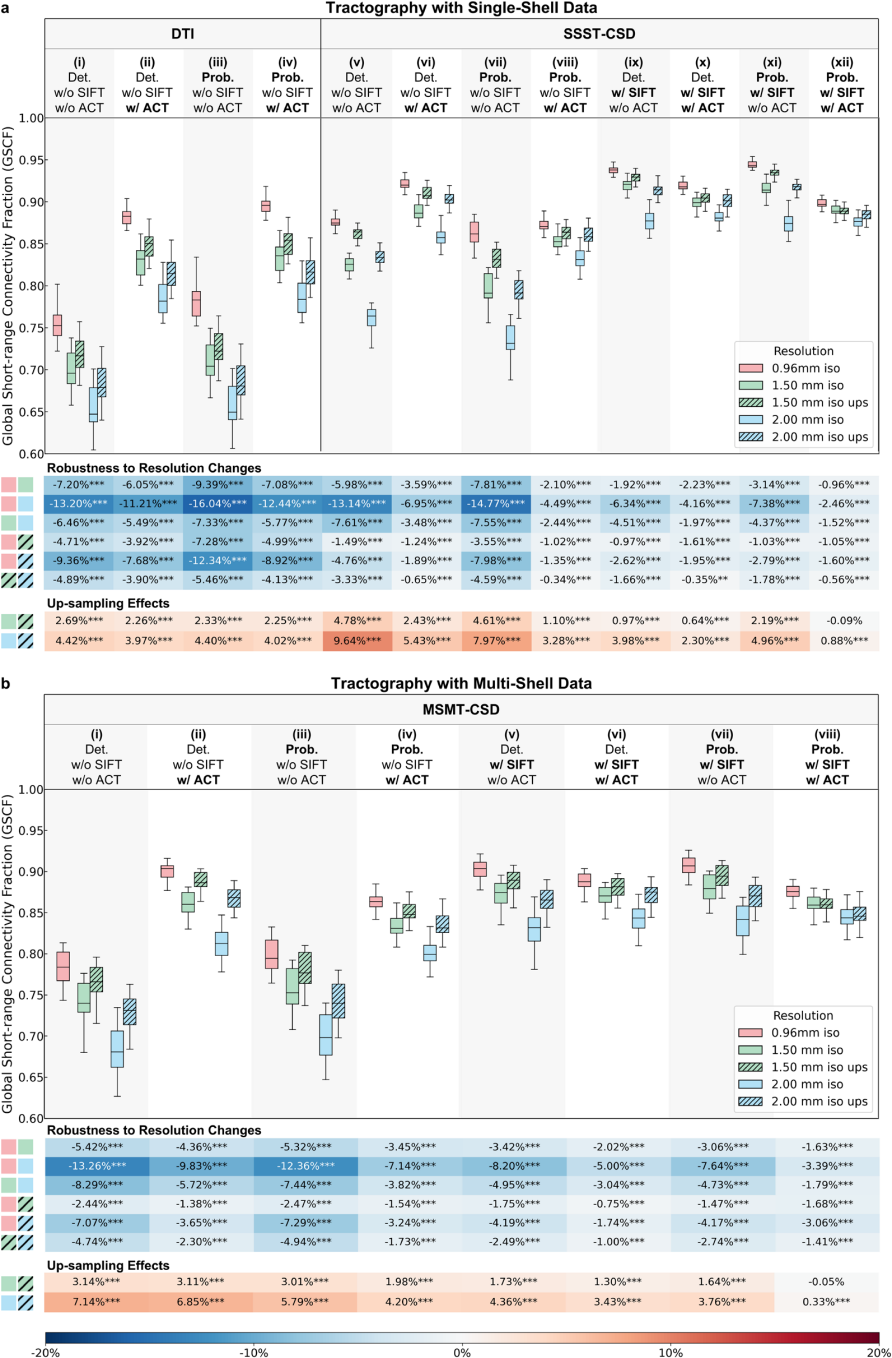
Prospectively acquired data GSCF. Box plots for GSCF from different tractography methods using single-shell (a) and multi-shell (b) data at three native spatial resolutions (red: 0.96 mm iso., green: 1.5 mm iso., blue: 2 mm iso.) and two nominally high 0.96 mm iso. resolution up-sampled from 1.5 mm and 2 mm iso. resolutions (hatched green: 1.5 mm up-sampled, hatched blue: 2 mm up-sampled) display the distribution (i.e., median, interquartile range, and range) of GSCF from 20 subjects in the upper panel. The tables show the relative GSCF differences at lower resolutions compared with higher resolutions (upper tables: robustness to resolution changes) and at up-sampled nominal 0.96 mm resolution compared with native resolutions (lower tables: up-sampling effects) (each row) for different tractography methods (each column), with asterisks denoting significance levels (^*^: p < 0.05, ^**^: p < 0.01, ^***^: p < 0.001). The color of each table cell indicates the magnitude and direction of the GSCF difference with a shared color bar at the bottom.

Lower spatial resolution consistently led to decreased GSCF (Fig. 5; Supplementary Fig. S4). The magnitude of this resolution-induced GSCF reduction varied across modeling and tracking methods. Regarding fiber modeling methods, DTI single-fiber models exhibited the largest decreases in GSCF (Fig. 5a, i–iv), while CSD-based crossing fiber models showed lower changes (Fig. 5a, v–xii, b).

Regarding tracking methods, probabilistic and deterministic algorithms showed comparable sensitivity to resolution changes for single-shell data (Fig. 5a, iii, iv, vii, viii, xi, xii vs. i, ii, v, vi, ix, x), while deterministic algorithms were more sensitive for multi-shell data using MSMT-CSD (Fig. 5b, i, ii, v, vi vs. iii, iv, vii, viii). Moreover, GSCF obtained using SIFT (Fig. 5a, ix–xii and b, v–viii) showed greater robustness to resolution changes than without using SIFT (Fig. 5a, v–viii and b, i–iv). Finally, methods lacking anatomical constraints (Fig. 5a, i, iii, v, vii, ix, xi and 5b, i, iii, v, vii) consistently showed larger GSCF reductions than methods incorporating ACT (Fig. 5a, ii, iv, vi, viii, x, xii and b, ii, iv, vi, viii).

Data up-sampling generally increased GSCF, partially offsetting the reduction caused by lower native resolution across most methods. However, for CSD-based probabilistic tracking incorporating both SIFT and ACT, the most commonly adopted tractography option, the compensatory effect was nearly negligible (Fig. 5a, xii and b, viii). Generally, tracking methods without using SIFT or ACT benefitted more from data up-sampling.

**Fig. 6.**
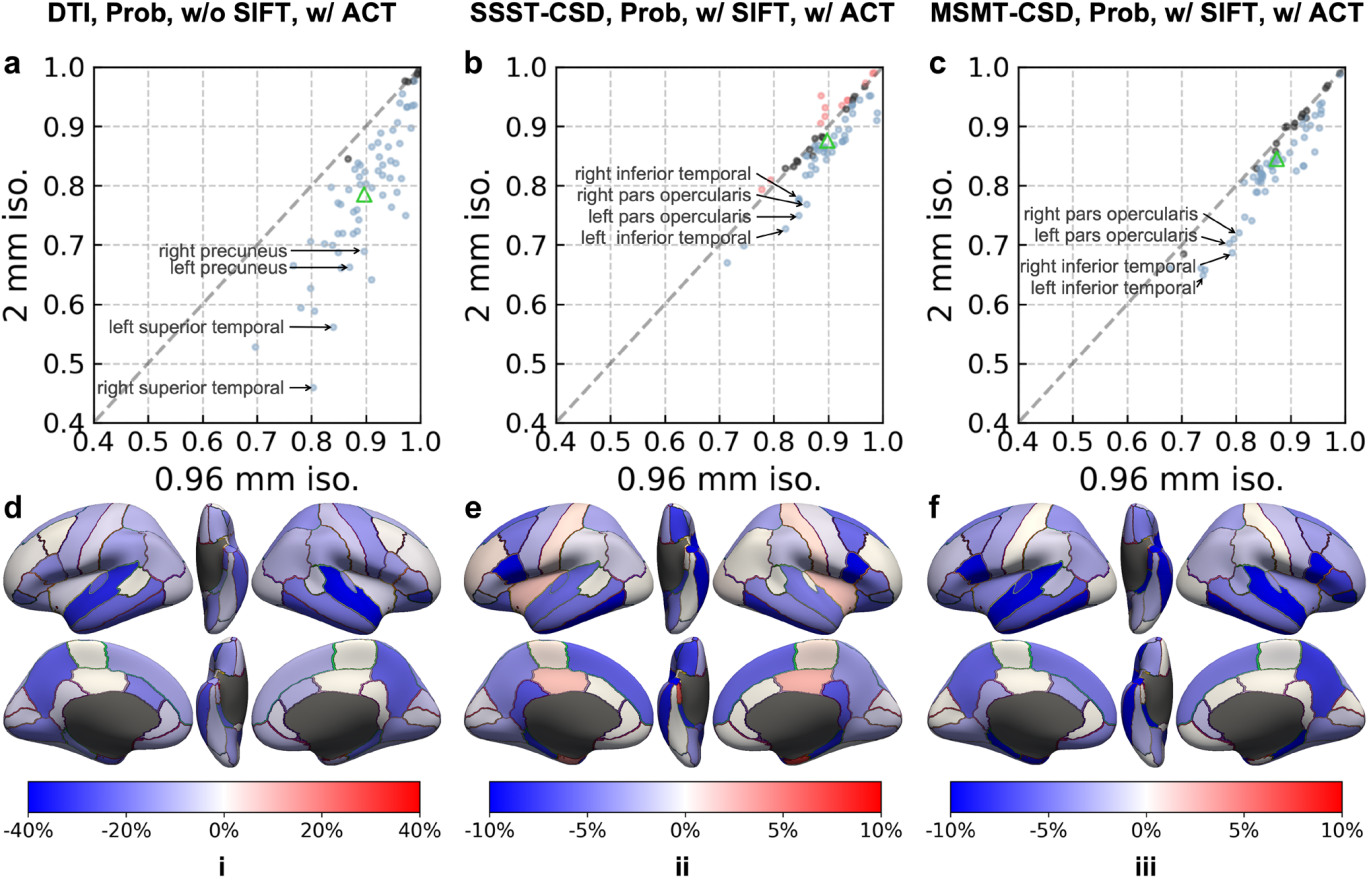
Prospectively acquired data RSCF. (a-c) Scatter plots comparing RSCF at high resolution (0.96 mm iso., x-axis) versus at low resolution (native 2 mm iso., y-axis) are displayed with each point representing a cortical region, color representing the difference and its significance (blue = decrease, red = increase, black = none), and the green triangle representing GSCF for anatomically constrained probabilistic tracking with different modeling and tracking options (i: DTI on low b-value single-shell data without SIFT, ii: SSST-CSD on high b-value single-shell data with SIFT, iii: MSMT-CSD on multi b-value data with SIFT). (d-f) The difference of RSCF at low resolution compared with RSCF at high resolution for each cortical region is displayed on inflated surfaces, with color and its intensity representing the pattern.

Lower spatial resolution reduced RSCF in most cortical regions, with the temporal lobe showing the greatest sensitivity, although the magnitude and distribution of this effect varied across tractography methods (Fig. 6; Supplementary Fig. S7; Table 3 for prospectively acquired data; and Supplementary Fig. S8; Supplementary Table S3 for retrospectively down-sampled data). Among the three representative pipelines shown in Fig. 6, DTI-based tractography was most affected, with 61 regions showing significant RSCF decreases (Fig. 6a), particularly in the superior temporal cortex and precuneus (Fig. 6d). SSST-CSD-based tractography was less affected, with 43 regions decreasing and 12 showing small increases (Fig. 6b), with the pars opercularis and inferior temporal cortex among the most sensitive regions (Fig. 6e). MSMT-CSD-based tractography showed an intermediate pattern, with 53 regions decreasing (Fig. 6c), most prominently in the pars opercularis and inferior temporal cortex (Fig. 6f). Spatial patterns for other tractography methods are shown in Supplementary Fig. S7.

**Table 3.** Spatial pattern of RSCF difference in prospectively acquired data.

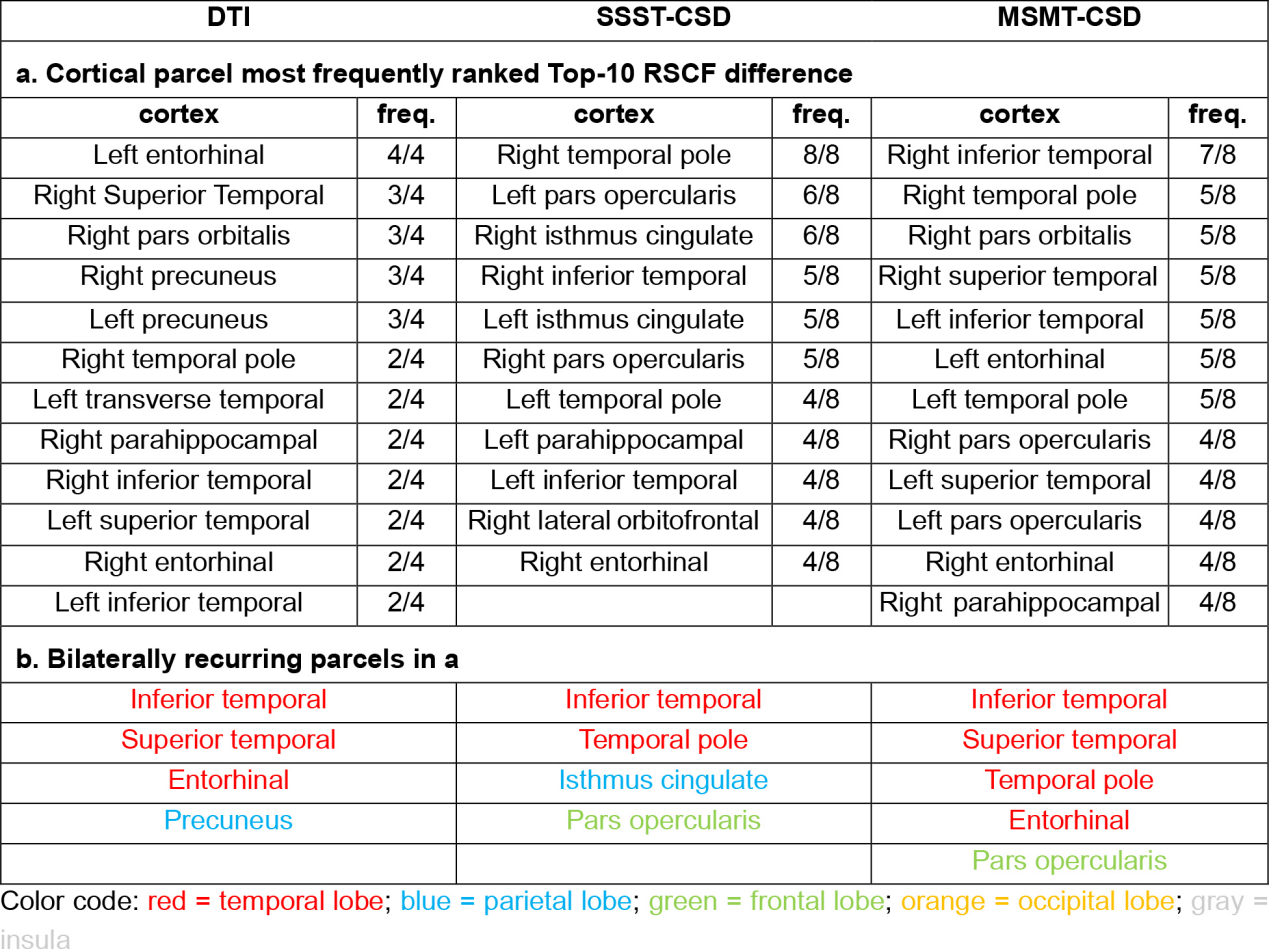

Table 3a summarizes the frequency with which each cortical region appeared among the Top-10 RSCF reductions across tractography methods using the same model fitting strategy (only regions with a frequency ≥50% are shown). Table 3b highlights regions where both left and right homologous structures were identified in Table 3a, with their respective lobes color encoded. Notably, temporal lobe regions (in red) make up the majority. A broadly consistent pattern was also found in the retrospectively down-sampled dataset (Supplementary Fig. S8; Supplementary Table S3).

## 4 Discussion

Our study systematically quantified the impact of spatial resolution on SAF reconstruction by comparing model fitting, tractography results, and connectivity-based quantitative metrics in MRtrix3. Degradation of the superficial WM architecture at lower resolution was observed in model fitting results, primarily attributable to increased partial volume effects (Fig. 3; Supplementary Fig. S2). Tractography revealed well-formed U-shaped fibers at all resolutions, though streamline counts decreased at lower resolutions (Fig. 4; Supplementary Fig. S3). Quantitative analyses further showed that lower resolution led to a decrease in global and regional SCF (Fig. 5; Supplementary Fig. S4), most pronounced in temporal lobe regions (Fig. 6; Table 3; Supplementary Figs. S7–S8; Supplementary Table S3).

Our study provides a systematic, quantitative evaluation of how dMRI spatial resolution and tractography methodology influence SAF reconstruction and structural connectivity estimation using MRtrix3 software. While the neuroimaging community generally accepts that higher spatial resolution improves the visualization of SAFs and often demonstrated this phenomenon qualitatively in studies showcasing new high-resolution dMRI sequences (Dong et al., 2025; Z. Li et al., 2026; Setsompop et al., 2018), systematic and quantitative supporting evidence has been lacking. Previous across-resolution comparisons suffered from several limitations, including the reliance on qualitative assessments without quantification (Song et al., 2014), the use of indirect comparisons across different datasets or cohorts that hinder direct interpretation (F. Zhang et al., 2024), or a restricted scope only evaluating a single tractography pipeline (Reveley et al., 2015; Song et al., 2014; F. Zhang et al., 2024). Our study confronts these gaps by providing quantitative assessments using connectivity-based metrics of GSCF, RSCF, and subsequent network topological properties such as small worldness (Supplementary Figs. S16 and S17) (Jiang et al., 2021; Zhou, 2017) and direct (i.e. within-subject) comparisons across 3 spatial resolutions and 20 distinct MRtrix3-based tractography pipelines, comprehensively evaluating critical factors including spatial resolution, data up-sampling, diffusion-encoding sensitivity, deterministic or probabilistic tracking, SIFT and ACT, on both prospectively acquired and retrospectively down-sampled multi-resolution data. Thanks to the state-of-the-art high-resolution dMRI sequence gSlider, the 0.96 mm iso. dMRI data and the 1.5 and 2 mm iso. data were acquired from 20 healthy individuals, providing a direct, across-resolution and quantitative comparison on prospectively acquired data, to our knowledge, for the first time. This comprehensive approach establishes a rigorous base for optimizing dMRI acquisition and processing strategies.

The fundamental reason that hampers the accurate reconstruction of SAFs at lower resolutions is the signal partial volume effects that degrades voxel-wise fiber modeling and delineation (Fig. 3; Supplementary Fig. S2). It primarily occurs through a complex inter-tissue and inter-fiber mixing of signals from GM, SAFs (tangential to the cortical fold), and long-range fibers entering the cortex (radial to the cortical fold) at the superficial WM layer. Generally, GM signal contamination reduces the FA or FOD integral, potentially causing premature streamline termination. Long-range fiber contamination increases the volume fraction of radial fibers, potentially reducing the representation of SAFs and increasing the possibility for erroneous fiber pathways, a well-studied “crossing-fiber” problem in the dMRI community (K. G. Schilling et al., 2017). Single-tissue, single-fiber methods such as DTI reconstruct only one principal orientation per voxel, producing the most visually continuous and coherent streamlines (Fig. 4a; Supplementary Fig. S3a). However, this angular simplification makes them more susceptible to partial volume effects, rendering them less robust to decreases in image resolution (Fig. 5a; -7.4% GSCF change from 0.96 to 1.5 mm iso., -13.1% GSCF change from 0.96 to 2 mm iso.) compared with the multi-fiber methods such as SSST-CSD (Fig. 5a; -3.4% GSCF change from 0.96 to 1.5 mm iso., -7.4% GSCF change from 0.96 to 2 mm iso.) and MSMT-CSD (Fig. 5b; -3.5% GSCF change from 0.96 to 1.5 mm iso., -8.2% GSCF change from 0.96 to 2 mm iso.). This suggests that resolution-dependent effects should be taken into consideration when comparing network metrics across studies and centers.

The choice of tracking strategies significantly influences the sensitivity of SAF estimation to dMRI spatial resolution. Probabilistic tracking (Fig. 5; -4.3% GSCF change from 0.96 to 1.5 mm iso., -8.7% GSCF change from 0.96 to 2 mm iso.) and deterministic tracking (Fig. 5; -4.1% GSCF change from 0.96 to 1.5 mm iso., -9.0% GSCF change from 0.96 to 2 mm iso.) showed comparable robustness to resolution decrease. Tractography without ACT and SIFT resulted in lower GSCF robustness across resolutions (Fig. 5; -6.2% GSCF change from 0.96 to 1.5 mm iso., -13.4% GSCF change from 0.96 to 2 mm iso.). Unconstrained seeding (uniform across the brain volume) places more seeds along long-range fibers than along SAFs, leading to disproportionate reconstruction of long-range fibers that overshadow SAFs (Smith et al., 2013). ACT may mitigate this problem by restricting seeding to the GM–WM interface (Smith et al., 2012), while SIFT refines tractograms by matching streamline density to FOD amplitude, removing redundant long-range fibers (Smith et al., 2013, 2015). Consequently, combining ACT and SIFT yielded the most robust GSCF across resolutions and likely the most biologically accurate SAF reconstruction and connectome estimation (Fig. 5; -1.7% GSCF change from 0.96 to 1.5 mm iso., -3.8% GSCF change from 0.96 to 2 mm iso.).

Resolution sensitivity in SAF reconstruction varies across cortical regions. The temporal lobe consistently demonstrates the greatest reduction in SAF reconstruction performance as resolution decreases (Fig. 6; Table 3; Supplementary Figs. S7–S8; Supplementary Table S3). This heightened sensitivity is likely attributed to the temporal lobe’s complex fiber architecture (Bajada et al., 2017), which increases modeling complexity, and its thinner SWM layer (K. G. Schilling et al., 2023), which exacerbates partial volume effects. This spatial pattern is crucial for characterizing SAF and its alterations in pathological conditions, particularly in Alzheimer’s disease research, as (1) the temporal lobe is among the earliest regions to exhibit amyloid deposition (Braak & Braak, 1996) and (2) SAFs connecting the temporal lobe show more pronounced microstructural alterations (S. Wang et al., 2025), underscoring their critical role. Additionally, the spatial pattern of SAF reconstruction varies across tractography methods, suggesting that different model fitting and tractography strategies may introduce varying degrees of bias at lower resolution. It also differs across datasets, indicating that SAF reconstruction may be influenced by imaging protocols (e.g., diffusion encoding schemes, readout durations), although potential effects of sample size cannot be ruled out.

Up-sampling low-resolution data partially recovers lost GSCF within the tractography pipelines evaluated in this study. For most tractography pipelines, up-sampling increased GSCF modestly above the native low-resolution baseline, yet it remained well below the native high-resolution benchmark. This effect was most pronounced for DTI-based methods, suggesting that up-sampling can partially mitigate partial volume effects in DTI, consistent with previous findings (Dyrby et al., 2014). For CSD-based methods, while the estimated FODs accurately represent the signal within each voxel, the reduced proportion of tangential fibers at lower resolutions poses challenges for tractography. Data up-sampling alleviated some of these difficulties, yet it typically failed to restore GSCF to native high-resolution levels (Dmitri et al., 2019). Notably, for the recommended and mostly widely used CSD probabilistic tracking equipped with both SIFT and ACT strategies, the initial fiber tracking was already robust (Fig. 5; -1.3% GSCF change from 0.96 to 1.5 mm iso., -2.9% GSCF change from 0.96 to 2 mm iso.), data up-sampling offered minimal additional improvement in GSCF. Instances of SAF streamline counts exceeding native high-resolution values after up-sampling (Fig. 4; Supplementary Fig. S3) may reflect interpolation-induced smoothing (Sommer et al., 2017) rather than better SAF reconstruction quality. This finding suggests that up-sampling cannot fully compensate for an insufficient native voxel size and the acquisition spatial resolution might be another critical factor that requires careful consideration when processing multi-center dMRI data (i.e., dMRI data harmonization (L. Ning et al., 2020)) in addition to diffusion-encoding sensitivity and direction.

Our findings are consistent across different definitions of SAF (Ouyang et al., 2017). Some studies included intra-regional connections as SAFs (K. G. Schilling et al., 2023), whereas others excluded them (Ouyang et al., 2016). Additionally, some studies applied length constraints to define SAFs (Gao et al., 2014; Guevara et al., 2017; K. G. Schilling et al., 2023). To test the robustness of our findings across different SAF definitions, we recalculated GSCF and RSCF by including intra-regional connections, imposing a 50-mm length threshold, or applying both criteria together. The results, as reflected in GSCF and RSCF, remained largely consistent (Supplementary Figs. S10–S15; Supplementary Tables S4–S9).

Based on our findings, we provide quantitative evidence supporting established methodological choices for optimized dMRI strategies in SAF reconstruction and characterization. First, high-resolution (preferably sub-millimeter) dMRI is recommended whenever feasible. Although higher spatial resolution entails an SNR penalty due to the resolution-SNR trade-off (Fig. 2; Supplementary Figs. S1 and S5), it nonetheless enhances SAF reconstruction. Our study demonstrated that sequences like gSlider achieved this within a practical timeframe (~30 minutes) for research, while alternative approaches including 3D multi-slab EPI (Engström & Skare, 2013), MB-MUSE (Bruce et al., 2017; Chen et al., 2013), SMSlab (Liu et al., 2023), EPTI (F. Wang et al., 2019), Romer-EPTI (Dong et al., 2025), and in-plane segmented 3D multi-slab sequences (Z. Li et al., 2026) offer comparable level high-fidelity sub-millimeter resolution. Furthermore, the advent of next-generation hardware, such as the CONNECTOME 2.0 (Huang et al., 2021; Ramos-Llordén et al., 2025), next-generation 7T scanners (Feinberg et al., 2023), along with clinically approved systems like the Siemens Cima.X (Kara et al., 2024), is making high-resolution dMRI acquisition increasingly feasible. Second, multi-shell data using a b-value higher than 2000 s/mm^2^ are preferable, since multi-tissue multi-fiber models such as MSMT-CSD are capable of separating different tissue and axon compartments and less prone to partial volume effects. When high-resolution acquisition is impractical or dMRI data are retrospectively analyzed, post-processing approaches less sensitive to resolution changes can help yield SAF results closer to those achieved with native high-resolution data. Based on the given acquisition parameters, the following processing approaches are recommended: for single-shell low b-value data (approximately b = 1000 s/mm²) in DTI, up-sample the dMRI data and use probabilistic tracking with anatomical constraints (i.e., Low B, Sing, Prob, w/o SIFT, w/ ACT) appear optimal; for single-shell medium-to-high b-value data (b = 2000 s/mm² or higher) and multi-shell data, use CSD-based probabilistic tracking with SIFT and ACT are optimal (i.e., High B, Xing, Prob, w/ SIFT, w/ ACT and Multi B, Xing, Prob, w/ SIFT, w/ ACT).

**Fig. 7.**
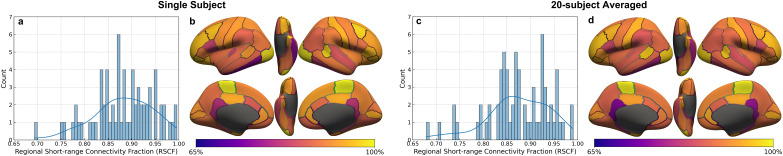
Distribution of RSCF across cortical regions. Histograms (a, c) and spatial maps of RSCF on the inflated cortical surface (b, d) for a representative subject (a, b) and the group average of 20 subjects (c, d) are shown.

Based on these optimized dMRI strategies for SAF reconstruction, we created the first human brain atlas of SAF fraction (Fig. 7), reconstructed from gSlider data of 20 healthy subjects acquired at 0.96 mm iso. resolution using CSD-based probabilistic tracking with SIFT and ACT. The results show that RSCF values exceed 0.65 across all brain regions, with most ranging from 0.82 to 0.96, further confirming the dominant role of SAFs in overall connectivity (Schüz & Miller, 2002). Among all regions, the paracentral cortex exhibits the highest SAF fraction, approaching 1, whereas the isthmus cingulate shows the lowest, falling below 0.7 (Supplementary Fig. S9). This atlas advances previous research on temporal changes in SAF fraction during brain development (Ouyang et al., 2016) by additionally revealing its spatial distribution in young adults (23.45 ± 1.80 years). The RSCF values in this atlas are higher than those derived using low-resolution imaging (2 mm iso.) and simpler tractography methods (DTI-based deterministic tracking without ACT) in this developmental study, emphasizing the importance of data acquisition and processing optimization.

Several limitations highlight avenues for future research. First, the SAF reconstructed from high-resolution data cannot be considered as a gold standard due to limitations such as reduced SNR, even though we used both prospectively acquired and retrospectively down-sampled data as complementary approaches. This major limitation highlights the need for future validation studies, potentially using high-quality, multi-resolution dMRI data obtained from very long scan times on post-mortem brains (Dyrby et al., 2014). Second, this study focused on healthy adults. Future work will examine whether the approach improves the sensitivity of SAF and connectivity-related biomarkers in patient populations, such as those with neurodegenerative diseases, as well as in both healthy and diseased children. Finally, while we evaluated widely used tractography algorithms in MRtrix3 for practical relevance (Gerussi et al., 2023; Grotheer et al., 2022; Lehman et al., 2020; Pruckner et al., 2023; Radwan et al., 2022; F. Zhang et al., 2022), future studies will incorporate emerging surface-based methods (Gahm & Shi, 2019; Y. Li et al., 2024; Nie et al., 2024; Shastin et al., 2022) to provide a more comprehensive understanding of resolution effects and potentially improve SAF reconstruction accuracy.

## 5 Conclusions

This study systematically quantified the impact of spatial resolution on SAF reconstruction and structural connectivity estimation using multi-resolution dMRI dataset for 20 healthy subjects using MRtrix3. Lower resolution results in SAF underestimation, particularly in temporal lobe regions, and the effect is most pronounced for simpler models (e.g., DTI) and basic tractography methods (without ACT or SIFT). Up-sampling data prior to tractography partially restore reconstruction accuracy within the evaluated pipelines. These findings provide quantitative evidence for SAF reconstruction and support established methodological preferences: high-resolution multi-shell acquisitions yield more accurate SAF reconstruction. When such data are unavailable, up-sampling coupled with DTI-based probabilistic tracking with ACT performs best for single-shell low b-value data, while CSD-based probabilistic tracking with SIFT and ACT is preferable for single-shell high b-value or multi-shell data. Furthermore, this study constructed the first atlas of the human brain RSCF, leveraging the recommended high-resolution dataset (0.96 mm iso.) and the CSD-based probabilistic tracking with SIFT and ACT, providing valuable knowledge for SAF research.

## Supplementary Material

Supplementary Material

## Data Availability

The prospectively acquired data are available on request from the corresponding author. The retrospectively down-sampled data from 20 subjects are provided by the Human Connectome Project WU-Minn Consortium and are all available via public database at https://www.humanconnectome.org. The codes for gradient nonlinearity correction are available at https://github.com/ksubramz/gradunwarp. The software used for data processing is also publicly available: FreeSurfer (https://surfer.nmr.mgh.harvard.edu/fswiki/DownloadAndInstall); FMRIB Software Library (FSL) (https://fsl.fmrib.ox.ac.uk/fsl/docs/#/install/index); MRtrix3 (https://www.mrtrix.org/download/); GRETNA (https://www.nitrc.org/frs/?group_id=668). The gSlider sequence and its reconstruction scripts can be accessed through the Collaborating to Commercialize and Publish (C2P) platform (https://www.nmr.mgh.harvard.edu/c2p).
