# Supplementary material for: Effects of diffusion MRI spatial resolution on human brain short-range association fiber reconstruction and structural connectivity estimation
Source: Imaging Neurosci (Camb). 2026 Jan 14;4:IMAG.a.1089. doi: 10.1162/IMAG.a.1089 (PMC12825271; doi:10.1162/IMAG.a.1089)
Supplement: Supplementary Material [file IMAG.a.1089_supp.pdf]

Effects of diffusion MRI spatial resolution on human brain short-range association fiber reconstruction and structural connectivity estimation

Jialan Zheng[1], Ziyu Li[2*], Wen Zhong[1], Ziang Wang[1], Zihan Li[1], Hongjia Yang[1], Mingxuan Liu[1], Xiaozhi Cao[3], Congyu Liao[4], David H. Salat[5, 6], Susie Y. Huang[5, 6], Qiyuan Tian[1*]

[1]School of Biomedical Engineering, Tsinghua University, Beijing, China;

[2]Oxford Centre for Integrative Neuroimaging, FMRIB, Nuffield Department of Clinical Neurosciences, University of Oxford, Oxford, United Kingdom;

[3]Department of Radiology, Stanford University, Stanford, CA, United States;

[4]Department of Radiology & Biomedical Imaging, University of California San Francisco, San Francisco, CA, United States;

[5]Athinoula A. Martinos Center for Biomedical Imaging, Department of Radiology, Massachusetts General Hospital, Charlestown, MA, United States;

[6]Harvard Medical School, Boston, MA, United States.

[*]Correspondence to: Qiyuan Tian, Ph.D., Center for Biomedical Imaging Research at Tsinghua University, 30 Shuangqing Road, Haidian District, Beijing, China, 100084. E-mail: qiyuantian@tsinghua.edu.cn. Ziyu Li, Ph.D., Oxford Centre for Integrative Neuroimaging, FMRIB, John Radcliffe Hospital, Oxford, United Kingdom, OX3 9DU. E-mail: ziyu.li@ndcn.ox.ac.uk.

Supplementary Information

Tractography Parameters

Table S1. Tracking parameters for prospectively acquired data

Method	Tracking Algorithm	Seed Number	Seed Location	Step Size (mm)	Angle (°)	Cutoff
Low B, Sing, w/o SIFT, Det, w/o ACT	Tensor_Det	5M	Whole brain	0.096	60	0.1
Low B, Sing, w/o SIFT, Det, w/ ACT	Tensor_Det	5M	GM-WM interface	0.096	60	0.05
Low B, Sing, w/o SIFT, Prob, w/o ACT	Tensor_Prob	5M	Whole brain	0.096	60	0.1
Low B, Sing, w/o SIFT, Prob, w/ ACT	Tensor_Prob	5M	GM-WM interface	0.096	60	0.05
High B, Xing, w/o SIFT, Det, w/o ACT	SD_Stream	5M	Whole brain	0.096	60	0.1
High B, Xing, w/o SIFT, Det, w/ ACT	SD_Stream	5M	GM-WM interface	0.096	60	0.05
High B, Xing, w/o SIFT, Prob, w/o ACT	iFOD2	5M	Whole brain	0.48	45	0.1
High B, Xing, w/o SIFT, Prob, w/ ACT	iFOD2	5M	GM-WM interface	0.48	45	0.05
High B, Xing, w/ SIFT, Det, w/o ACT	SD_Stream	5M	Whole brain	0.096	60	0.1
High B, Xing, w/ SIFT, Det, w/ ACT	SD_Stream	5M	GM-WM interface	0.096	60	0.05
High B, Xing, w/ SIFT, Prob, w/o ACT	iFOD2	5M	Whole brain	0.48	45	0.1
High B, Xing, w/ SIFT, Prob, w/ ACT	iFOD2	5M	GM-WM interface	0.48	45	0.05
Multi B, Xing, w/o SIFT, Det, w/o ACT	SD_Stream	5M	Whole brain	0.096	60	0.1
Multi B, Xing, w/o SIFT, Det, w/ ACT	SD_Stream	5M	GM-WM interface	0.096	60	0.05
Multi B, Xing, w/o SIFT, Prob, w/o ACT	iFOD2	5M	Whole brain	0.48	45	0.1
Multi B, Xing, w/o SIFT, Prob, w/ ACT	iFOD2	5M	GM-WM interface	0.48	45	0.05
Multi B, Xing, w/ SIFT, Det, w/o ACT	SD_Stream	5M	Whole brain	0.096	60	0.05
Multi B, Xing, w/ SIFT, Det, w/ ACT	SD_Stream	5M	GM-WM interface	0.096	60	0.05
Multi B, Xing, w/ SIFT, Prob, w/o ACT	iFOD2	5M	Whole brain	0.48	45	0.1
Multi B, Xing, w/ SIFT, Prob, w/ ACT	iFOD2	5M	GM-WM interface	0.48	45	0.05

Abbreviations: Low B = single-shell with low b-value; High B = single-shell with high b-value; Multi B = multiple shells with both low and high b-values; Sing = single-fiber model; Xing = crossing-fiber model; Det = deterministic; Prob = probabilistic; SIFT = spherical-deconvolution informed filtering of tractograms; ACT = anatomically-constrained tractography; GM = gray matter; WM = white matter.

Table S2. Tracking parameters for retrospectively down-sampled data

Method	Tracking Algorithm	Seed Number	Seed Location	Step Size (mm)	Angle (°)	Cutoff
Low B, Sing, w/o SIFT, Det, w/o ACT	Tensor_Det	5M	Whole brain	0.125	60	0.1
Low B, Sing, w/o SIFT, Det, w/ ACT	Tensor_Det	5M	GM-WM interface	0.125	60	0.05
Low B, Sing, w/o SIFT, Prob, w/o ACT	Tensor_Prob	5M	Whole brain	0.125	60	0.1
Low B, Sing, w/o SIFT, Prob, w/ ACT	Tensor_Prob	5M	GM-WM interface	0.125	60	0.05
High B, Xing, w/o SIFT, Det, w/o ACT	SD_Stream	5M	Whole brain	0.125	60	0.1
High B, Xing, w/o SIFT, Det, w/ ACT	SD_Stream	5M	GM-WM interface	0.125	60	0.05
High B, Xing, w/o SIFT, Prob, w/o ACT	iFOD2	5M	Whole brain	0.625	45	0.1
High B, Xing, w/o SIFT, Prob, w/ ACT	iFOD2	5M	GM-WM interface	0.625	45	0.05
High B, Xing, w/ SIFT, Det, w/o ACT	SD_Stream	5M	Whole brain	0.125	60	0.1
High B, Xing, w/ SIFT, Det, w/ ACT	SD_Stream	5M	GM-WM interface	0.125	60	0.05
High B, Xing, w/ SIFT, Prob, w/o ACT	iFOD2	5M	Whole brain	0.625	45	0.1
High B, Xing, w/ SIFT, Prob, w/ ACT	iFOD2	5M	GM-WM interface	0.625	45	0.05
Multi B, Xing, w/o SIFT, Det, w/o ACT	SD_Stream	5M	Whole brain	0.125	60	0.1
Multi B, Xing, w/o SIFT, Det, w/ ACT	SD_Stream	5M	GM-WM interface	0.125	60	0.05
Multi B, Xing, w/o SIFT, Prob, w/o ACT	iFOD2	5M	Whole brain	0.625	45	0.1
Multi B, Xing, w/o SIFT, Prob, w/ ACT	iFOD2	5M	GM-WM interface	0.625	45	0.05
Multi B, Xing, w/ SIFT, Det, w/o ACT	SD_Stream	5M	Whole brain	0.125	60	0.05
Multi B, Xing, w/ SIFT, Det, w/ ACT	SD_Stream	5M	GM-WM interface	0.125	60	0.05
Multi B, Xing, w/ SIFT, Prob, w/o ACT	iFOD2	5M	Whole brain	0.625	45	0.1
Multi B, Xing, w/ SIFT, Prob, w/ ACT	iFOD2	5M	GM-WM interface	0.625	45	0.05

Abbreviations: Low B = single-shell with low b-value; High B = single-shell with high b-value; Multi B = multiple shells with both low and high b-values; Sing = single-fiber model; Xing = crossing-fiber model; Det = deterministic; Prob = probabilistic; SIFT = Spherical-deconvolution Informed Filtering of Tractograms; ACT = anatomically-constrained tractography; GM = gray matter; WM = white matter.

Results for Retrospectively Down-sampled Data

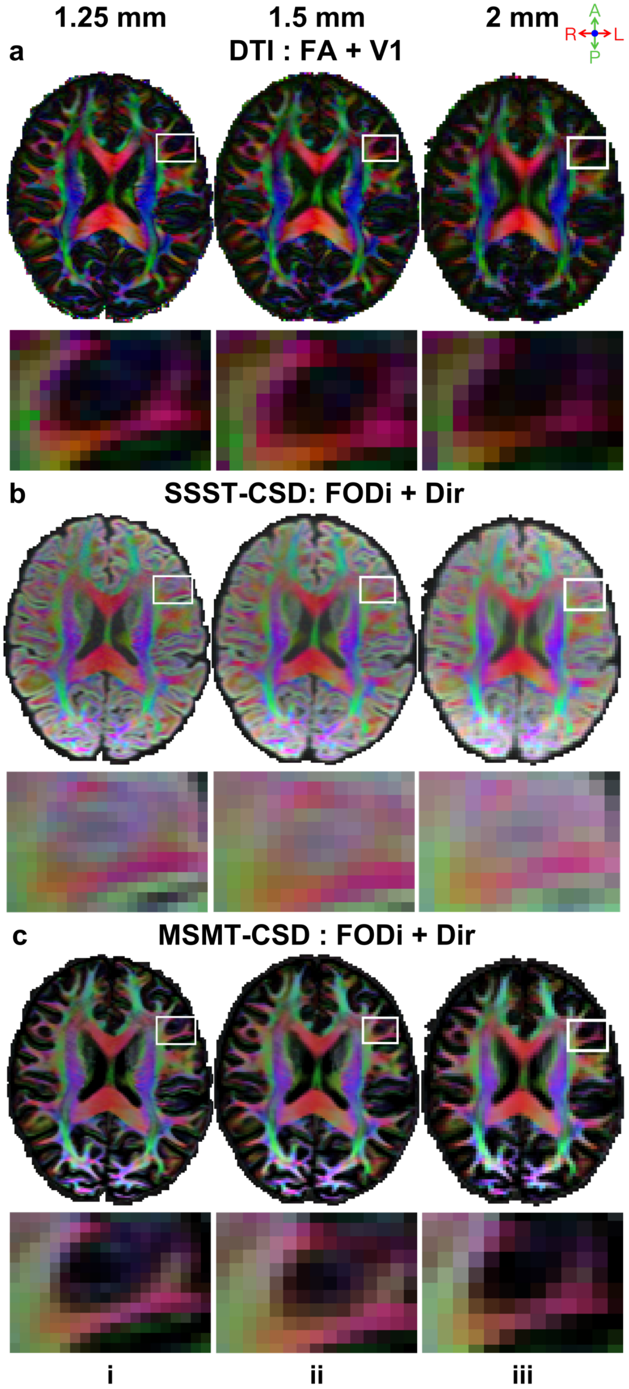

Figure S1. Retrospectively down-sampled data quality. Axial slices from a representative subject with fitted results from three diffusion models (i: DTI, ii: SSST-CSD, iii: MSMT-CSD) across three resolutions (i: 1.25 mm, ii: 1.5 mm, iii: 2 mm) are displayed. The fractional anisotropy (FA) map color-encoded by primary vector (V1) (a) and the fiber orientation distribution integral (FODi) map color-encoded by the overall fiber direction (b, c) are shown for DTI and CSD methods (red: left-right; green: anterior-posterior; blue: superior-inferior), respectively.

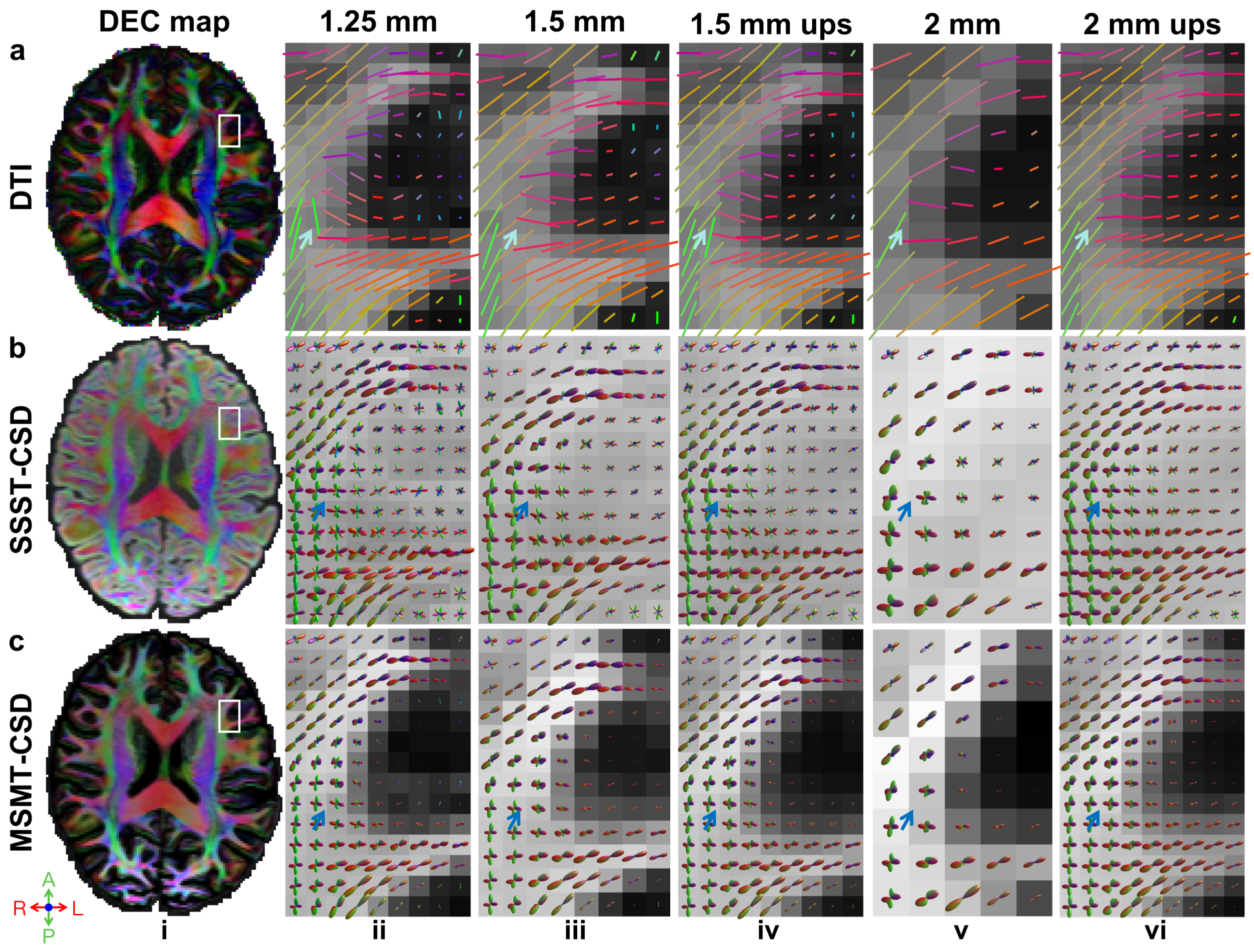

Figure S2. Fiber orientations from retrospectively down-sampled data. Primary fiber direction encoded color (DEC) maps (red: left-right; green: anterior-posterior; blue: superior-inferior) at 1.25 mm iso. resolution from a representative subject (i) are displayed for three methods including DTI (a, i), SSST-CSD (b, i), and MSMT-CSD (c, i), with a region of interest (white boxes) containing gyri, subcortical white matter, and their interface shown in enlarged views overlaid on FA (a), and FOD integral (b, c) maps across three different spatial resolutions (ii: 1.25 mm, iii: 1.5 mm, v: 2 mm) and two nominally high 1.25 mm iso. resolution up-sampled from 1.5 mm and 2 mm iso. resolutions (iv: 1.5 mm up-sampled, vi: 2 mm up-sampled). Blue arrows highlight a region with short-range association fibers.

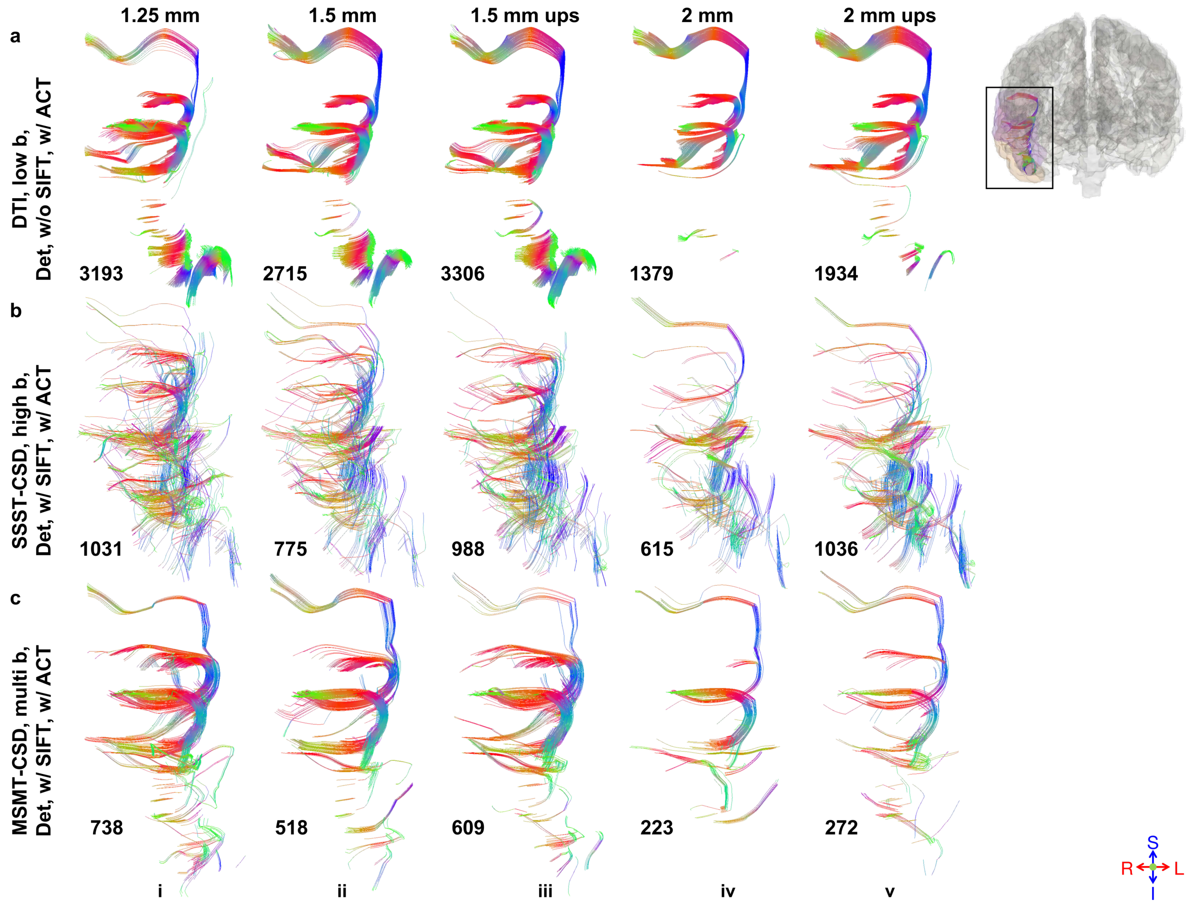

Figure S3. Retrospectively down-sampled data tractography results. U-shaped SAFs connecting the right middle and superior temporal gyri (black box), extracted from whole-brain tractograms reconstructed using the same number of seeds and three modeling methods (a: DTI on low b-value single-shell data, b: SSST-CSD on high b-value single-shell data with SIFT, c: MSMT-CSD on multi b-value data with SIFT), as well as deterministic, anatomically-constrained tractography (ACT) across three different native spatial resolutions (i: 1.25 mm, ii: 1.5 mm, iv: 2 mm) and two nominally high 1.25 mm iso. resolution up-sampled from 1.5 and 2 mm iso. resolutions (iii: 1.5 mm up-sampled, v: 2 mm up-sampled) are displayed. Each tract is annotated (lower-left) with either the number of streamlines (a) or the sum of streamline weights (b and c).

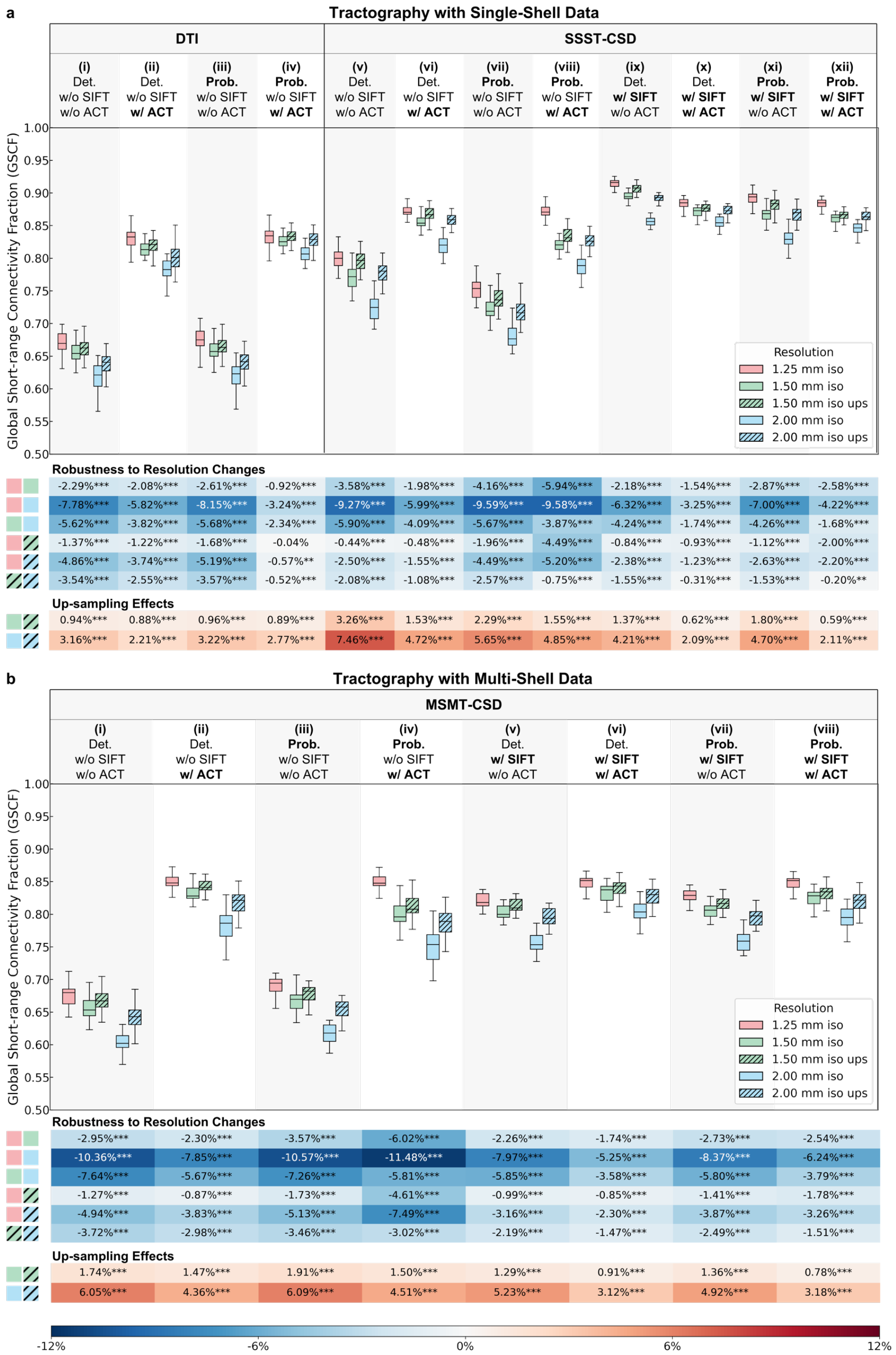

Figure S4. Retrospectively down-sampled data GSCF. Box plots for GSCF from different tractography methods using single-shell (a) and multi-shell (b) data at three native spatial resolutions (red: 1.25 mm iso., green: 1.5 mm iso., blue: 2 mm iso.) and two nominally high 1.25 mm iso. resolution up-sampled from 1.5 mm and 2 mm iso. resolutions (hatched green: 1.5 mm up-sampled, hatched blue: 2 mm up-sampled) display the distribution (i.e., median, interquartile range, and range) of GSCF from 20 subjects in the upper panel. The tables show the relative GSCF differences at lower resolutions compared to higher resolutions (upper tables: robustness to resolution changes) and at up-sampled nominal 1.25 mm resolution compared to native resolutions (lower tables: up-sampling effects) (each row) for different tractography methods (each column), with asterisks denoting significance levels (*: $p < 0.05$, **: $p < 0.01$, ***: $p < 0.001$). The color of each table cell indicates the magnitude and direction of the GSCF difference with a shared color-bar at the bottom.

Temporal Signal-to-Noise Ratio

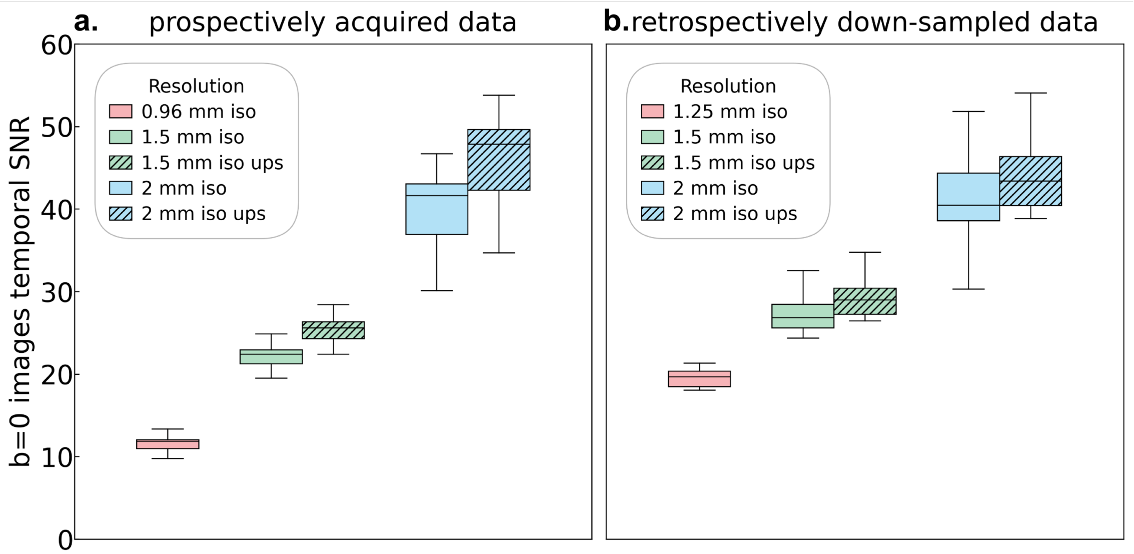

Figure S5. Temporal signal-to-noise ratio (SNR). Box plots for SNR of prospectively acquired data (a) and retrospectively down-sampled data (b) at three native spatial resolutions (red: highest resolution, 0.96 mm iso. for prospectively acquired data or 1.25 mm iso. for retrospectively down-sampled data; green: 1.5 mm iso.; blue: 2 mm iso.) and two nominally high resolutions up-sampled from 1.5 mm and 2 mm iso. resolutions (hatched green: 1.5 mm up-sampled, hatched blue: 2 mm up-sampled) display the distribution (i.e., median, interquartile range, and range) of SNR from 20 subjects in each dataset.

Temporal signal-to-noise ratio (SNR) exhibited a clear decreasing trend with increasing spatial resolution, consistent with the well-known trade-off between spatial resolution and SNR. Up-sampling improved the effective SNR, likely because the interpolation process attenuated noise while preserving relatively stable signal levels.

Selection of Superficial White Matter Layer

Masks of the gray matter-white matter interface (GMWMI; Figure S6, ii-vi, pink) were generated using the "5tt2gmwmi" command of MRtrix3. Within the region between two adjacent gyri, the layer of voxels closest to the underlying white matter was selected as an estimate of the superficial white matter (SWM) layer (Figure S6, ii-vi, dark blue). The mean fractional anisotropy (FA) was then calculated within this SWM layer mask for each resolution.

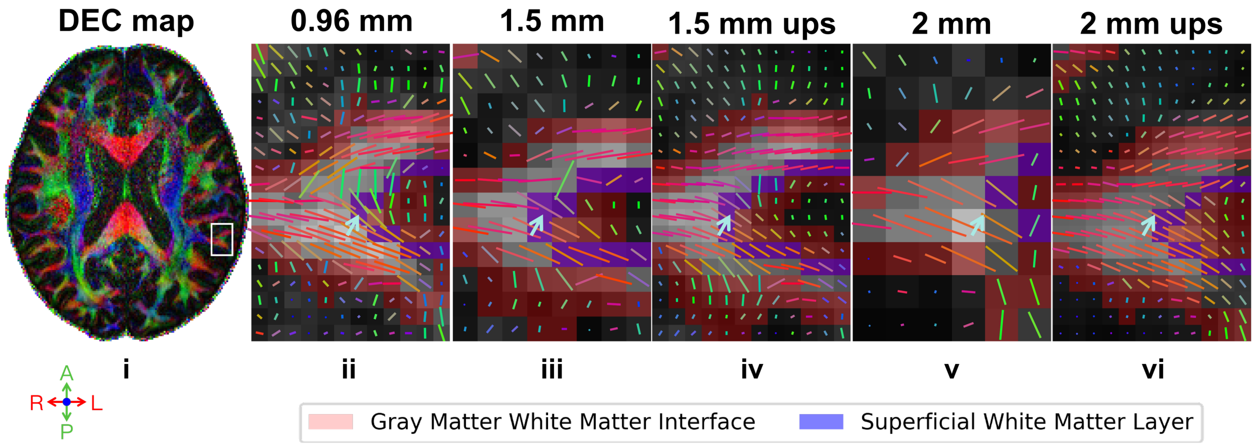

Figure S6. Superficial white matter selection. Primary fiber direction encoded color (DEC) maps (red: left-right; green: anterior-posterior; blue: superior-inferior) at 0.96 mm iso. resolution from a representative subject (i) are displayed for DTI model fitting, with a region of interest (white boxes) containing gyri, subcortical white matter, and their interface shown in enlarged views overlaid on FA maps across three different native spatial resolutions (ii: 0.96 mm, iii: 1.5 mm, v: 2 mm) and two nominally high 0.96 mm iso. resolution up-sampled from 1.5 mm and 2 mm iso. resolutions (iv: 1.5 mm up-sampled, vi: 2 mm up-sampled). Blue arrows highlight a region with short-range association fibers. Pink overlays indicate the gray matter-white matter interface mask, while dark blue overlays highlight the superficial white matter layer, defined as the voxels closest to the underlying white matter between adjacent gyri.

Spatial Pattern for RSCF Differences

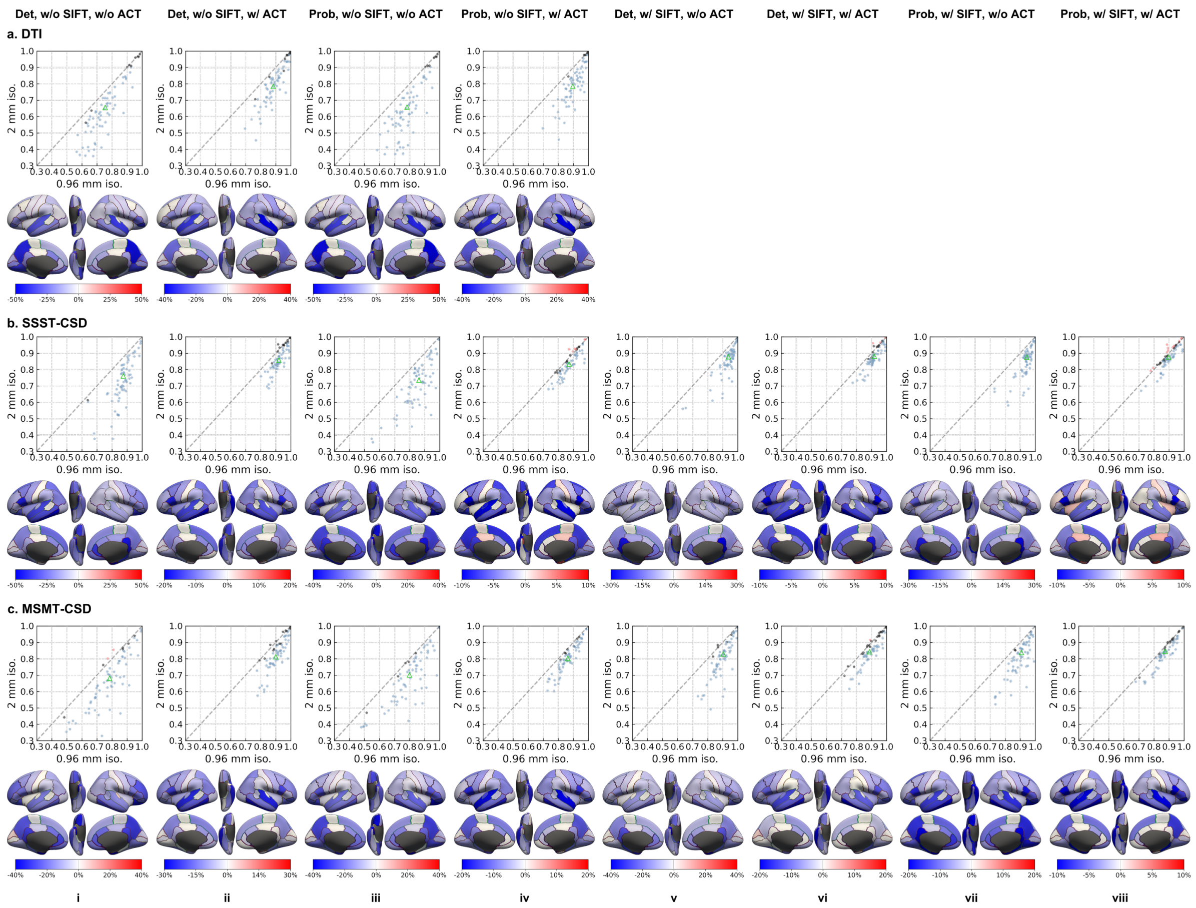

Figure S7. Prospectively acquired data RSCF. Scatter plots comparing RSCF at high resolution (0.96 mm iso., x-axis) versus at low resolution (native 2 mm iso., y-axis) are displayed with each point representing a cortical region, color representing the difference and its significance (blue = decrease, red = increase, black = none), and the green triangle representing GSCF for tractography methods annotated at the top of each column with different modeling and tracking options (a: DTI on low b-value single-shell data without SIFT, b: SSST-CSD on high b-value single-shell data with SIFT, c: MSMT-CSD on multi b-value data with SIFT). The difference of RSCF at low resolution compared to RSCF at high resolution for each cortical region is displayed on inflated surfaces, with color and its intensity representing the pattern.

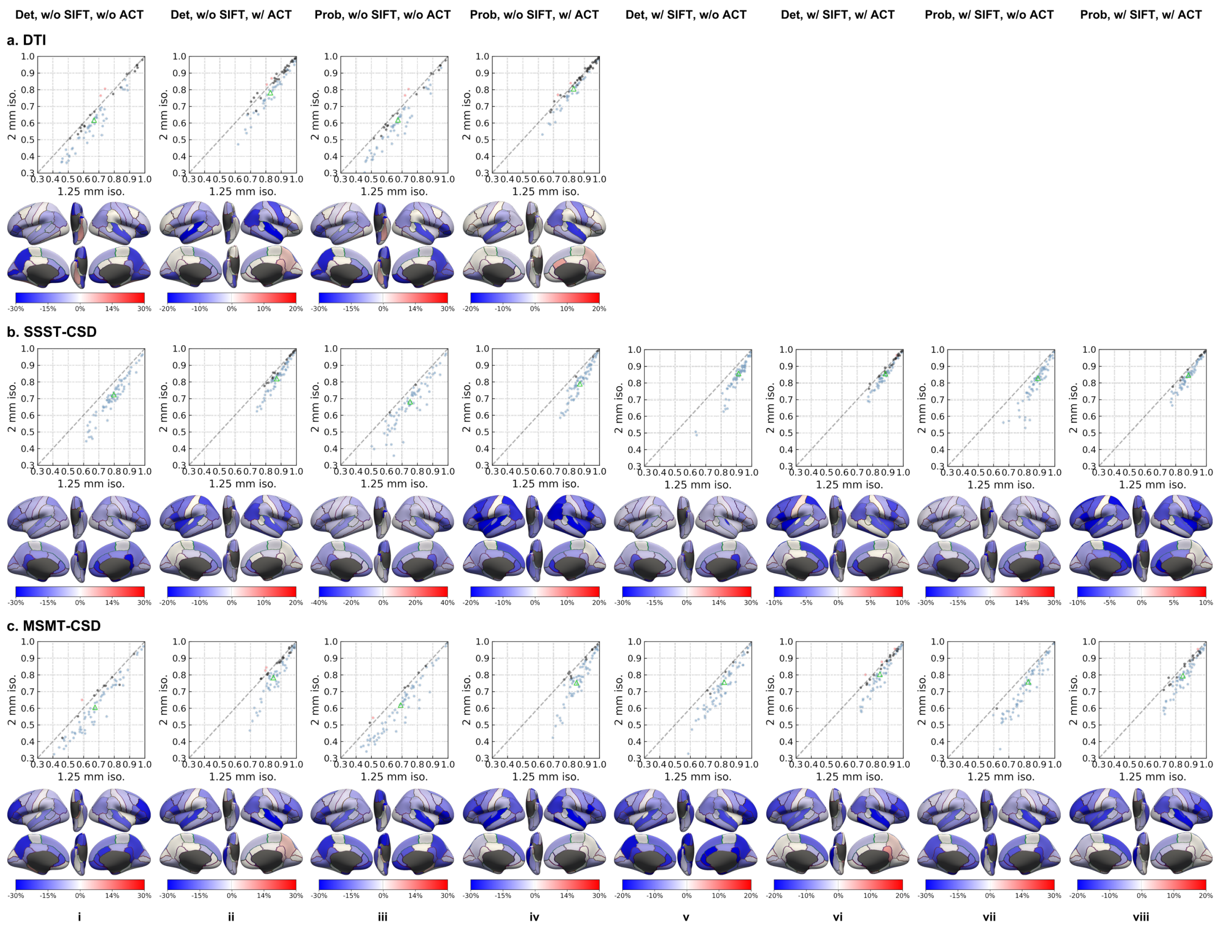

Figure S8. Retrospectively down-sampled data RSCF. Scatter plots comparing RSCF at high resolution (1.25 mm iso., x-axis) versus at low resolution (native 2 mm iso., y-axis) are displayed with each point representing a cortical region, color representing the difference and its significance (blue = decrease, red = increase, black = none), and the green triangle representing GSCF for tractography methods annotated at the top of each column with different modeling and tracking options (a: DTI on low b-value single-shell data without SIFT, b: SSST-CSD on high b-value single-shell data with SIFT, c: MSMT-CSD on multi b-value data with SIFT). The difference of RSCF at low resolution compared to RSCF at high resolution for each cortical region is displayed on inflated surfaces, with color and its intensity representing the pattern.

Table S3. Spatial pattern of RSCF difference in retrospectively down-sampled data.

a. Cortical region most frequently ranked Top-10 RSCF difference

DTI		SSST-CSD		MSMT-CSD	
**cortex**	**freq.**	**cortex**	**freq.**	**cortex**	**freq.**
Left temporal pole	4/4	Right pars opercularis	6/8	Right superior temporal	6/8
Left entorhinal	3/4	Right temporal pole	4/8	Right inferior temporal	6/8
Right transverse temporal	3/4	Right inferior temporal	4/8	Right temporal pole	4/8
Right temporal pole	2/4	Left pars opercularis	4/8	Right transverse temporal	4/8
Right superior temporal	2/4	Right isthmus cingulate	4/8	Right pars opercularis	4/8
Right inferior parietal	2/4	Right rostral anterior cingulate	4/8	Left precuneus	4/8
Right medial orbitofrontal	2/4	Left superior parietal	4/8	Left inferior temporal	4/8
Right precentral	2/4	Left insula	4/8	Left pars opercularis	4/8
Left superior temporal	2/4			Left temporal pole	4/8
Right entorhinal	2/4			Left superior temporal	4/8
Left lingual	2/4			Left rostral middle frontal	4/8
				Right rostral middle frontal	4/8

b. Bilaterally recurring regions in a.

DTI	SSST-CSD	MSMT-CSD
Temporal pole	Pars opercularis	Superior temporal
Superior temporal		Inferior temporal
Entorhinal		Temporal pole
		Pars opercularis
		Rostral middle frontal

Color key: red = temporal lobe; blue = parietal lobe; green = frontal lobe; orange = occipital lobe; gray = insula

Cortical Regions with the Highest or Lowest RSCF

The paracentral cortex exhibited the highest RSCF, approaching 1, with predominantly SAF connections to neighboring regions, including the precentral gyrus, postcentral gyrus, precuneus, and posterior cingulate (Figure S9 a). Additionally, some fibers entered the cingulum to reach frontal areas, consistent with previous studies (Saygi et al., 2023).

The isthmus cingulate cortex exhibited the lowest RSCF, falling below 0.7, with some fibers entering the cingulum and extending to the precuneus, para hippocampal, and lingual regions, while others projecting to broader areas such as the superior parietal, entorhinal, and frontal regions, consistent with previous studies (Ricchi et al., 2025; Yh et al., 2021).

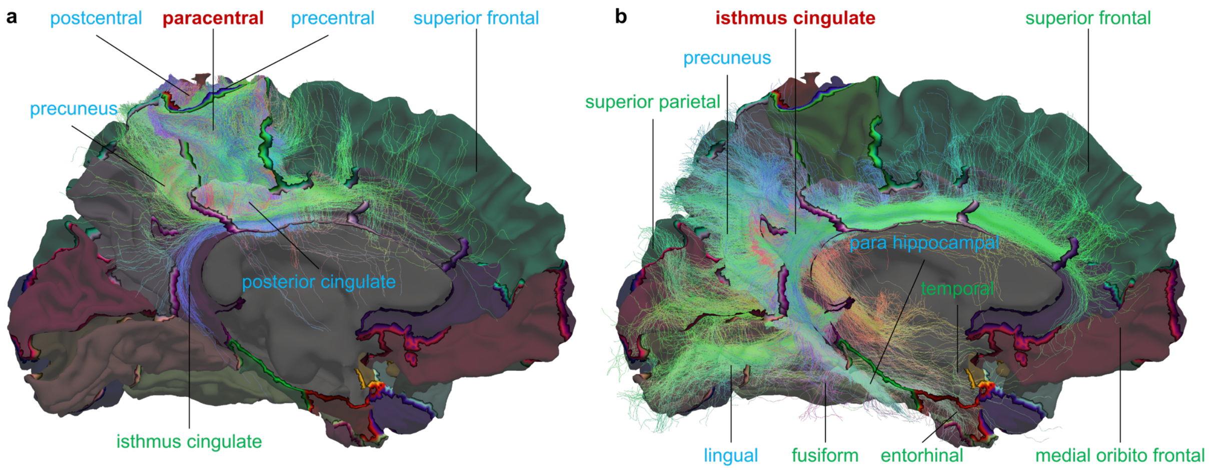

Figure S9. Association fibers originating from left paracentral and isthmus cingulate cortex. All association fibers originating from the left paracentral cortex (a) or the left isthmus cingulate cortex (b) were extracted from a whole-brain tractogram generated using CSD-based probabilistic tractography with SIFT and ACT. Visualization is shown for a representative subject at 0.96 mm iso. resolution. The gray matter-white matter interface surface was contracted by 1.5 mm and displayed for anatomical context, with different cortical regions shown in different colors and delineated with bold outlines. The cortex of origin is highlighted in red, while regions strongly connected to it are annotated in blue for adjacent regions and green for non-adjacent regions.

<u>Alternative SAF Definitions and GSCF/RSCF Results</u>

In addition to the SAF definition described in Sections 2.3-2.5, several alternative formulations of GSCF/RSCF were employed to assess the generalizability of our findings.

Variation 1 (including intra-region connections). Streamlines with both endpoints in the same cortical region ($i = j$) within the structural connectivity matrix were included. Equation (1) was therefore updated as:

$$\omega_{i,j} = \begin{cases} \mathrm{NOS}(i,j) & \text{if same hemisphere} \\ 0 & \text{otherwise} \end{cases} \tag{S1}$$

so that the self-connections were considered as SAFs. The adjacency matrix was adjusted ($A(i,i) = 1$) accordingly. All other steps for computing GSCF and RSCF remained unchanged. The results, as reflected in GSCF and RSCF, for both datasets are shown below.

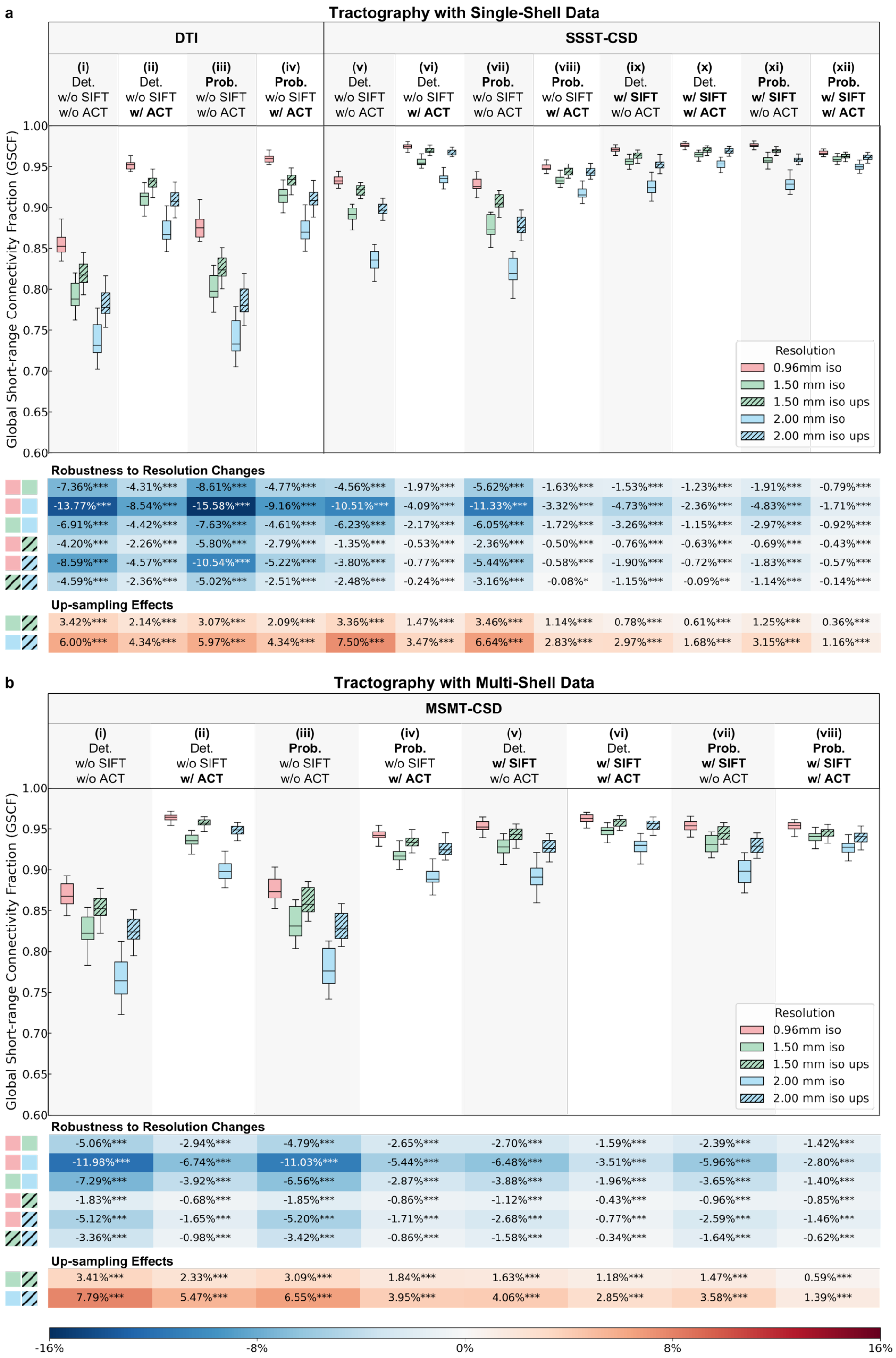

Figure S10. Prospectively acquired data Variation 1 GSCF. Box plots for GSCF from different tractography methods using single-shell (a) and multi-shell (b) data at three native spatial resolutions (red: 0.96 mm iso., green: 1.5 mm iso., blue: 2 mm iso.) and two nominally high 0.96 mm iso. resolution up-sampled from 1.5 mm and 2 mm iso. resolutions (hatched green: 1.5 mm up-sampled, hatched blue: 2 mm up-sampled) display the distribution (i.e., median, interquartile range, and range) of GSCF from 20 subjects in the upper panel. The tables show the relative GSCF differences at lower resolutions compared to higher resolutions (upper tables: robustness to resolution changes) and at up-sampled nominal 0.96 mm resolution compared to native resolutions (lower tables: up-sampling effects) (each row) for different tractography methods (each column), with asterisks denoting significance levels (*: $p < 0.05$, **: $p < 0.01$, ***: $p < 0.001$). The color of each table cell indicates the magnitude and direction of the GSCF difference with a shared color-bar at the bottom.

Table S4. Spatial pattern of Variation 1 RSCF difference in prospectively acquired data.

a. Cortical region most frequently ranked Top-10 RSCF difference

DTI		SSST-CSD		MSMT-CSD	
**cortex**	**freq.**	**cortex**	**freq.**	**cortex**	**freq.**
Right superior temporal	3/4	Left isthmus cingulate	8/8	Right temporal pole	6/8
Right parahippocampal	3/4	Right temporal pole	7/8	Right pars orbitalis	6/8
Right pars orbitalis	3/4	Right isthmus cingulate	7/8	Right pars opercularis	6/8
Left superior temporal	3/4	Left parahippocampal	6/8	Left temporal pole	6/8
Left transverse temporal	3/4	Left pars opercularis	6/8	Left isthmus cingulate	6/8
Right temporal pole	2/4	Right rostral anterior cingulate	6/8	Left pars opercularis	5/8
Left caudal anterior cingulate	2/4	Left temporal pole	5/8	Right parahippocampal	5/8
Left entorhinal	2/4	Right parahippocampal	5/8	Right isthmus cingulate	4/8
Right entorhinal	2/4	Right entorhinal	4/8	Left entorhinal	4/8
Right inferior temporal	2/4	Right pars opercularis	4/8	Left inferior temporal	4/8
Left inferior temporal	2/4	Left inferior temporal	4/8	Right entorhinal	4/8
Left pars orbitalis	2/4				
Left middle temporal	2/4				

b. Bilaterally recurring regions in a.

DTI	SSST-CSD	MSMT-CSD
Superior temporal	Temporal pole	Temporal pole
Inferior temporal	Parahippocampal	Entorhinal
Entorhinal	Isthmus cingulate	Isthmus cingulate
Pars orbitails	Pars opercularis	Pars opercularis

Color key: red = temporal lobe; blue = parietal lobe; green = frontal lobe; orange = occipital lobe; gray = others (limbic lobe, insula, etc.)

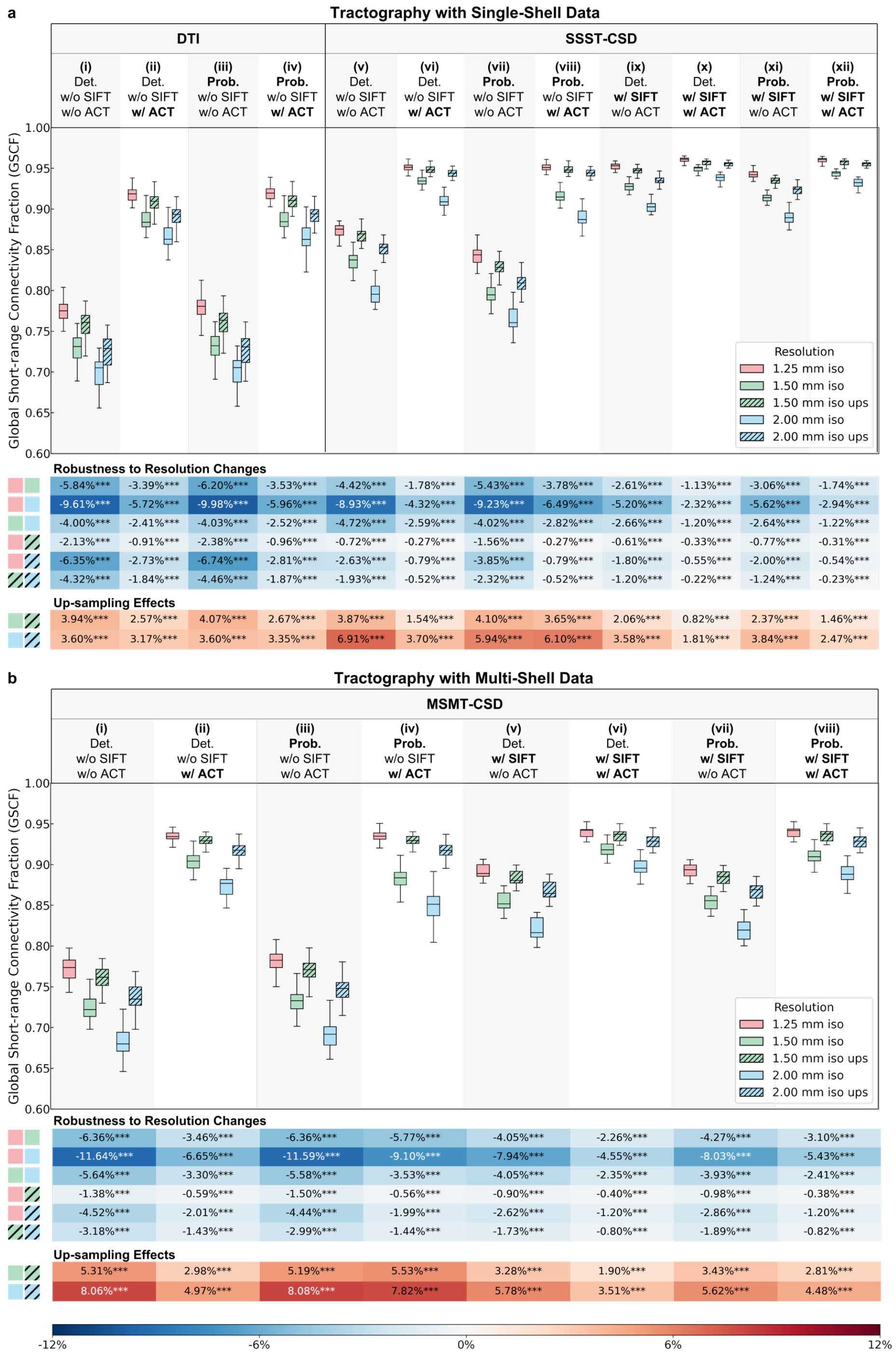

Figure S11. Retrospectively down-sampled data Variation 1 GSCF. Box plots for GSCF from different tractography methods using single-shell (a) and multi-shell (b) data at three native spatial resolutions (red: 1.25 mm iso., green: 1.5 mm iso., blue: 2 mm iso.) and two nominally high 1.25 mm iso. resolution up-sampled from 1.5 mm and 2 mm iso. resolutions (hatched green: 1.5 mm up-sampled, hatched blue: 2 mm up-sampled) display the distribution (i.e., median, interquartile range, and range) of GSCF from 20 subjects in the upper panel. The tables show the relative GSCF differences at lower resolutions compared to higher resolutions (upper tables: robustness to resolution changes) and at up-sampled nominal 1.25 mm resolution compared to native resolutions (lower tables: up-sampling effects) (each row) for different tractography methods (each column), with asterisks denoting significance levels (*: $p < 0.05$, **: $p < 0.01$, ***: $p < 0.001$). The color of each table cell indicates the magnitude and direction of the GSCF difference with a shared color-bar at the bottom.

Table S5. Spatial pattern of Variation 1 RSCF difference in retrospectively down-sampled data.

a. Cortical region most frequently ranked Top-10 RSCF difference

DTI		SSST-CSD		MSMT-CSD	
**cortex**	**freq.**	**cortex**	**freq.**	**cortex**	**freq.**
Left temporal pole	4/4	Right rostral anterior cingulate	8/8	Right superior temporal	6/8
Right transverse temporal	3/4	Right pars opercularis	6/8	Right pars opercularis	5/8
Left entorhinal	3/4	Left rostral anterior cingulate	5/8	Right pars orbitalis	5/8
Right superior temporal	3/4	Left isthmus cingulate	5/8	Right pars triangularis	5/8
Right temporal pole	2/4	Right isthmus cingulate	4/8	Left pars opercularis	5/8
Right precentral	2/4	Right insula	4/8	Right transverse temporal	4/8
Left middle temporal	2/4	Left insula	4/8	Left insula	4/8
Left caudal anterior cingulate	2/4	Left temporal pole	4/8	Right temporal pole	4/8
Right inferior parietal	2/4	Right temporal pole	4/8	Right inferior parietal	4/8
Right medial orbito frontal	2/4	Left pars opercularis	4/8	Right entorhinal	4/8
Left transverse temporal	2/4			Right inferior temporal	4/8
Right entorhinal	2/4			Left temporal pole	4/8
Left superior temporal	2/4				

b. Bilaterally recurring regions in a.

DTI	SSST-CSD	MSMT-CSD
Superior temporal	Temporal pole	Temporal pole
Temporal pole	Isthmus cingulate	Pars opercularis
Transverse temporal	Pars opercularis	
Entorhinal	Rostral anterior cingulate	
	Insula	

Color key: red = temporal lobe; blue = parietal lobe; green = frontal lobe; orange = occipital lobe; gray = others (limbic lobe, insula, etc.)

Variation 2 (imposing a streamline length threshold). Streamlines have to satisfy both the adjacency condition $A(i,j) = 1$ and an arch length threshold $L < l$ in order to be counted as SAFs. Length-filtered structural connectivity matrix W^l was built as:

$$\omega^l{}_{i,j} = \begin{cases} NOS^l(i,j) & \text{if same hemisphere and } i \neq j \\ 0 & \text{otherwise} \end{cases} \quad \text{(S2)}$$

where $NOS^l(i,j)$ represented the number of streamlines connecting region i and region j and satisfying $L < l$. GSCF and RSCF were then recomputed using the length-constrained definition, replacing Equations (2) and (3), as:

$$\text{GSCF} = \frac{\sum_{i,j \text{ s.t. } A(i,j)=1} \omega^l{}_{i,j}}{\sum_{i,j} \omega_{i,j}}; \quad \text{(S3)}$$

$$\text{RSCF}_k = \frac{\sum_{j \text{ s.t. } A(k,j)=1} \omega^l{}_{k,j}}{\sum_{j} \omega_{k,j}}. \quad \text{(S4)}$$

A moderate length threshold $l = 50$ mm was adopted (Schilling et al., 2023) and related results are reported as below.

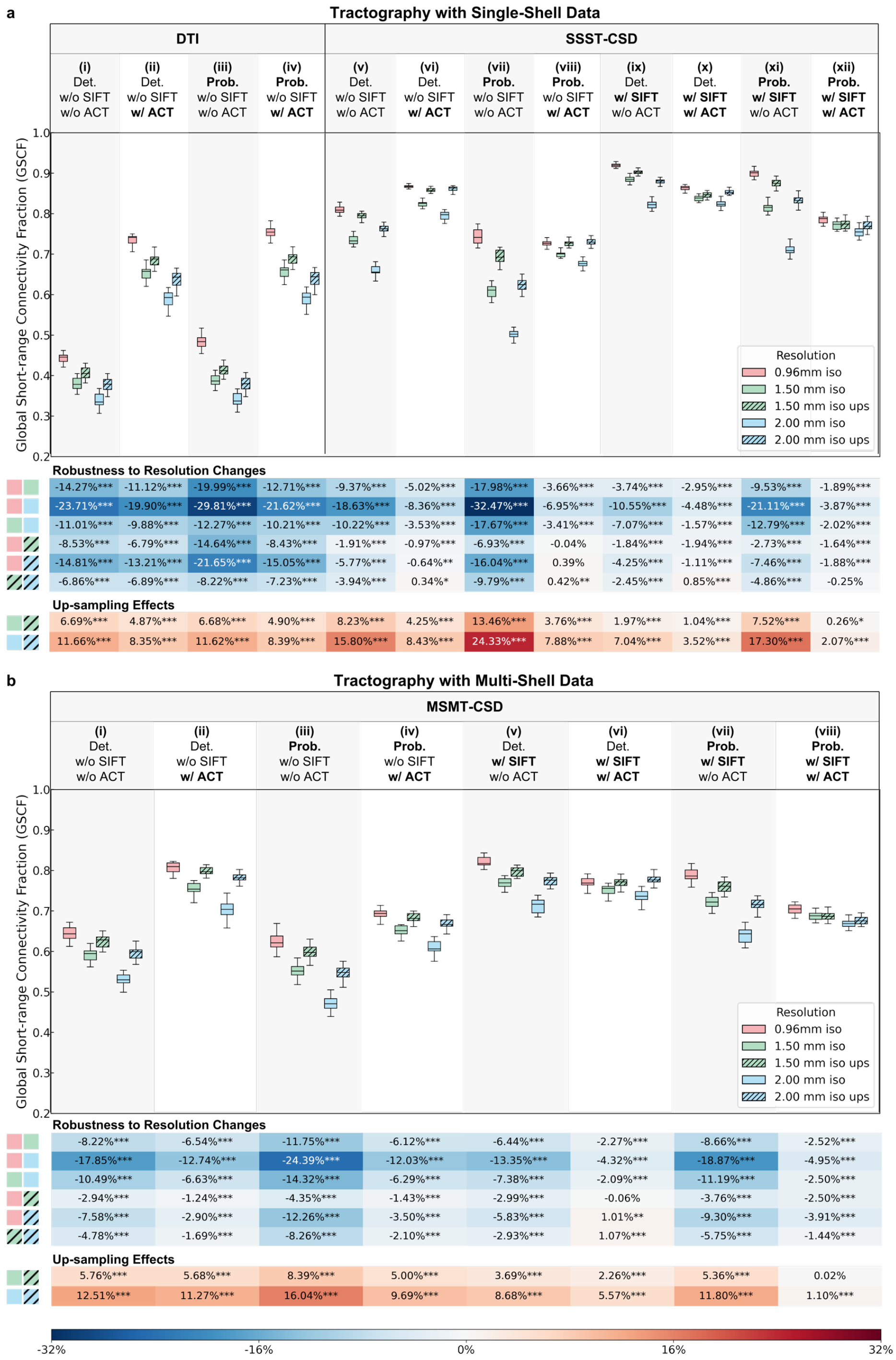

Figure S12. Prospectively acquired data Variation 2 GSCF. Box plots for GSCF from different tractography methods using single-shell (a) and multi-shell (b) data at three native spatial resolutions (red: 0.96 mm iso., green: 1.5 mm iso., blue: 2 mm iso.) and two nominally high 0.96 mm iso. resolution up-sampled from 1.5 mm and 2 mm iso. resolutions (hatched green: 1.5 mm up-sampled, hatched blue: 2 mm up-sampled) display the distribution (i.e., median, interquartile range, and range) of GSCF from 20 subjects in the upper panel. The tables show the relative GSCF differences at lower resolutions compared to higher resolutions (upper tables: robustness to resolution changes) and at up-sampled nominal 0.96 mm resolution compared to native resolutions (lower tables: up-sampling effects) (each row) for different tractography methods (each column), with asterisks denoting significance levels (*: $p < 0.05$, **: $p < 0.01$, ***: $p < 0.001$). The color of each table cell indicates the magnitude and direction of the GSCF difference with a shared color-bar at the bottom.

Table S6. Spatial pattern of Variation 2 RSCF difference in prospectively acquired data.

a. Cortical region most frequently ranked Top-10 RSCF difference

DTI		SSST-CSD		MSMT-CSD	
**cortex**	**freq.**	**cortex**	**freq.**	**cortex**	**freq.**
Left entorhinal	4/4	Left pars opercularis	6/8	Left inferior temporal	7/8
Right superior temporal	3/4	Left inferior temporal	6/8	Right inferior temporal	7/8
Right middle temporal	3/4	Right inferior temporal	5/8	Right superior temporal	6/8
Right pars orbitalis	3/4	Left parahippocampal	5/8	Left middle temporal	6/8
Left superior temporal	3/4	Right middle temporal	4/8	Left superior temporal	6/8
Right entorhinal	2/4	Right pars opercularis	4/8	Right pars opercularis	5/8
Right inferior temporal	2/4	Left middle temporal	4/8	Right middle temporal	5/8
Left middle temporal	2/4	Right lateral orbitofrontal	4/8	Left entorhinal	5/8
Left transverse temporal	2/4			Right entorhinal	4/8
Left inferior temporal	2/4			Left pars opercularis	4/8

b. Bilaterally recurring regions in a.

DTI	SSST-CSD	MSMT-CSD
Superior temporal	Middle temporal	Superior temporal
Middle temporal	Inferior temporal	Middle temporal
Inferior temporal	Pars opercularis	Inferior temporal
Entorhinal		Entorhinal
		Pars opercularis

Color key: red = temporal lobe; blue = parietal lobe; green = frontal lobe; orange = occipital lobe; gray = others (limbic lobe, insula, etc.)

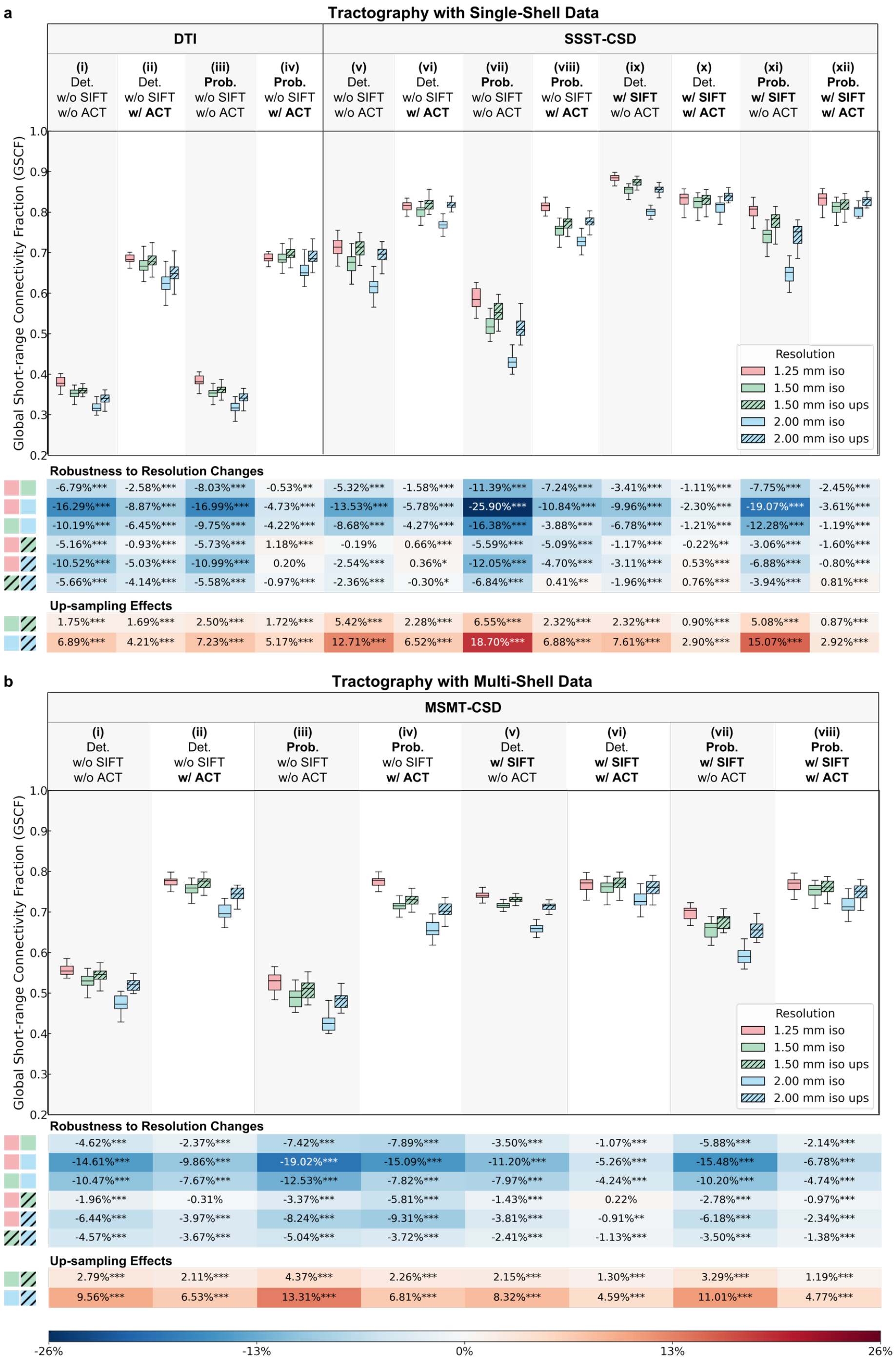

Figure S13. Retrospectively down-sampled data Variation 2 GSCF. Box plots for GSCF from different tractography methods using single-shell (a) and multi-shell (b) data at three native spatial resolutions (red: 1.25 mm iso., green: 1.5 mm iso., blue: 2 mm iso.) and two nominally high 1.25 mm iso. resolution up-sampled from 1.5 mm and 2 mm iso. resolutions (hatched green: 1.5 mm up-sampled, hatched blue: 2 mm up-sampled) display the distribution (i.e., median, interquartile range, and range) of GSCF from 20 subjects in the upper panel. The tables show the relative GSCF differences at lower resolutions compared to higher resolutions (upper tables: robustness to resolution changes) and at up-sampled nominal 1.25 mm resolution compared to native resolutions (lower tables: up-sampling effects) (each row) for different tractography methods (each column), with asterisks denoting significance levels (*: $p < 0.05$, **: $p < 0.01$, ***: $p < 0.001$). The color of each table cell indicates the magnitude and direction of the GSCF difference with a shared color-bar at the bottom.

Table S7. Spatial pattern of Variation 2 RSCF difference in retrospectively down-sampled data.

a. Cortical region most frequently ranked Top-10 RSCF difference

DTI		SSST-CSD		MSMT-CSD	
**cortex**	**freq.**	**cortex**	**freq.**	**cortex**	**freq.**
Right superior temporal	3/4	Left pars opercularis	5/8	Right superior temporal	7/8
Left temporal pole	3/4	Right insula	4/8	Right pars opercularis	6/8
Right transverse temporal	2/4	Right temporal pole	4/8	Right inferior temporal	6/8
Right rostral anterior cingulate	2/4	Right inferior temporal	4/8	Left inferior temporal	5/8
Right temporal pole	2/4	Right superior temporal	4/8	Left pars opercularis	5/8
Right medial orbitofrontal	2/4	Left insula	4/8	Left superior temporal	5/8
Left transverse temporal	2/4	Left superior temporal	4/8	Right rostral middle frontal	5/8
Right middle temporal	2/4	Right caudal middle frontal	4/8	Right temporal pole	4/8
Right precentral	2/4			Right middle temporal	4/8
Left entorhinal	2/4			Left rostral middle frontal	4/8
Left lingual	2/4			Left temporal pole	4/8
Left superior temporal	2/4				
Right inferior parietal	2/4				

b. Bilaterally recurring regions in a.

DTI	SSST-CSD	MSMT-CSD
Superior temporal	Superior temporal	Superior temporal
Temporal pole	Caudal middle frontal	Temporal pole
Transverse temporal	Insula	Inferior temporal
		Pars opercularis
		Rostral middle frontal

Color key: red = temporal lobe; blue = parietal lobe; green = frontal lobe; orange = occipital lobe; gray = others (limbic lobe, insula, etc.)

Variation 3 (combining both intra-region inclusion and length thresholding). This variation simultaneously includes intra-regional streamlines, as in Variation 1, and applies the length threshold, as in Variation 2 Therefore, the length-constrained connectivity matrix W^l that included intra-cortical connections was constructed as:

$$\omega^l{}_{i,j} = \begin{cases} NOS^l(i,j) & \text{if same hemisphere} \\ 0 & \text{otherwise} \end{cases}, \quad \text{(S5)}$$

and GSCF and RSCF were calculated accordingly to Equations (S3) and (S4). A length threshold $l = 50$ mm was adopted related results are reported as below.

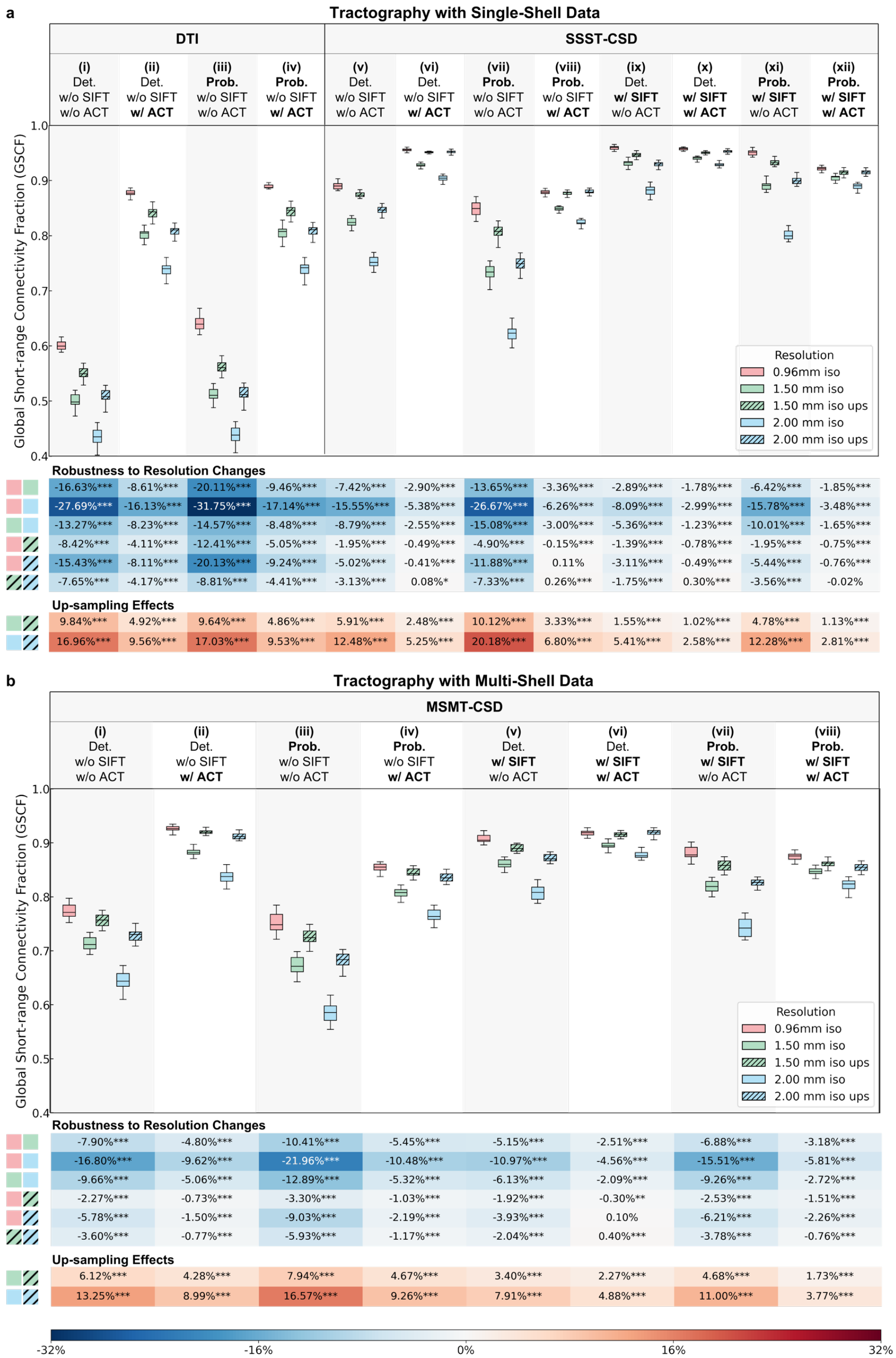

Figure S14. Prospectively acquired data Variation 3 GSCF. Box plots for GSCF from different tractography methods using single-shell (a) and multi-shell (b) data at three native spatial resolutions (red: 0.96 mm iso., green: 1.5 mm iso., blue: 2 mm iso.) and two nominally high 0.96 mm iso. resolution up-sampled from 1.5 mm and 2 mm iso. resolutions (hatched green: 1.5 mm up-sampled, hatched blue: 2 mm up-sampled) display the distribution (i.e., median, interquartile range, and range) of GSCF from 20 subjects in the upper panel. The tables show the relative GSCF differences at lower resolutions compared to higher resolutions (upper tables: robustness to resolution changes) and at up-sampled nominal 0.96 mm resolution compared to native resolutions (lower tables: up-sampling effects) (each row) for different tractography methods (each column), with asterisks denoting significance levels (*: $p < 0.05$, **: $p < 0.01$, ***: $p < 0.001$). The color of each table cell indicates the magnitude and direction of the GSCF difference with a shared color-bar at the bottom.

Table S8. Spatial pattern of Variation 3 RSCF difference in prospectively acquired data.

a. Cortical region most frequently ranked Top-10 RSCF difference

DTI		SSST-CSD		MSMT-CSD	
**cortex**	**freq.**	**cortex**	**freq.**	**cortex**	**freq.**
Right superior temporal	3/4	Left isthmus cingulate	7/8	Right pars opercularis	7/8
Right pars orbitalis	3/4	Right isthmus cingulate	7/8	Right temporal pole	6/8
Left superior temporal	3/4	Left parahippocampal	6/8	Left pars opercularis	6/8
Right middle temporal	3/4	Left pars opercularis	6/8	Right middle temporal	5/8
Right entorhinal	2/4	Right pars opercularis	6/8	Left inferior temporal	5/8
Left supramarginal	2/4	Right rostral anterior cingulate	5/8	Left middle temporal	5/8
Right pars opercularis	2/4	Right parahippocampal	5/8	Right inferior temporal	5/8
Right superior parietal	2/4	Left inferior temporal	4/8	Left parahippocampal	4/8
Left entorhinal	2/4	Right temporal pole	4/8	Left temporal pole	4/8
Left inferior temporal	2/4	Right entorhinal	4/8	Right entorhinal	4/8
Left middle temporal	2/4			Left entorhinal	4/8
Left pars opercularis	2/4				

b. Bilaterally recurring regions in a.

DTI	SSST-CSD	MSMT-CSD
Superior temporal	Parahippocampal	Temporal pole
Middle temporal	Isthmus cingulate	Middle temporal
Entorhinal	Pars opercularis	Inferior temporal
Pars opercularis		Entorhinal
		Pars opercularis

Color key: red = temporal lobe; blue = parietal lobe; green = frontal lobe; orange = occipital lobe; gray = others (limbic lobe, insula, etc.)

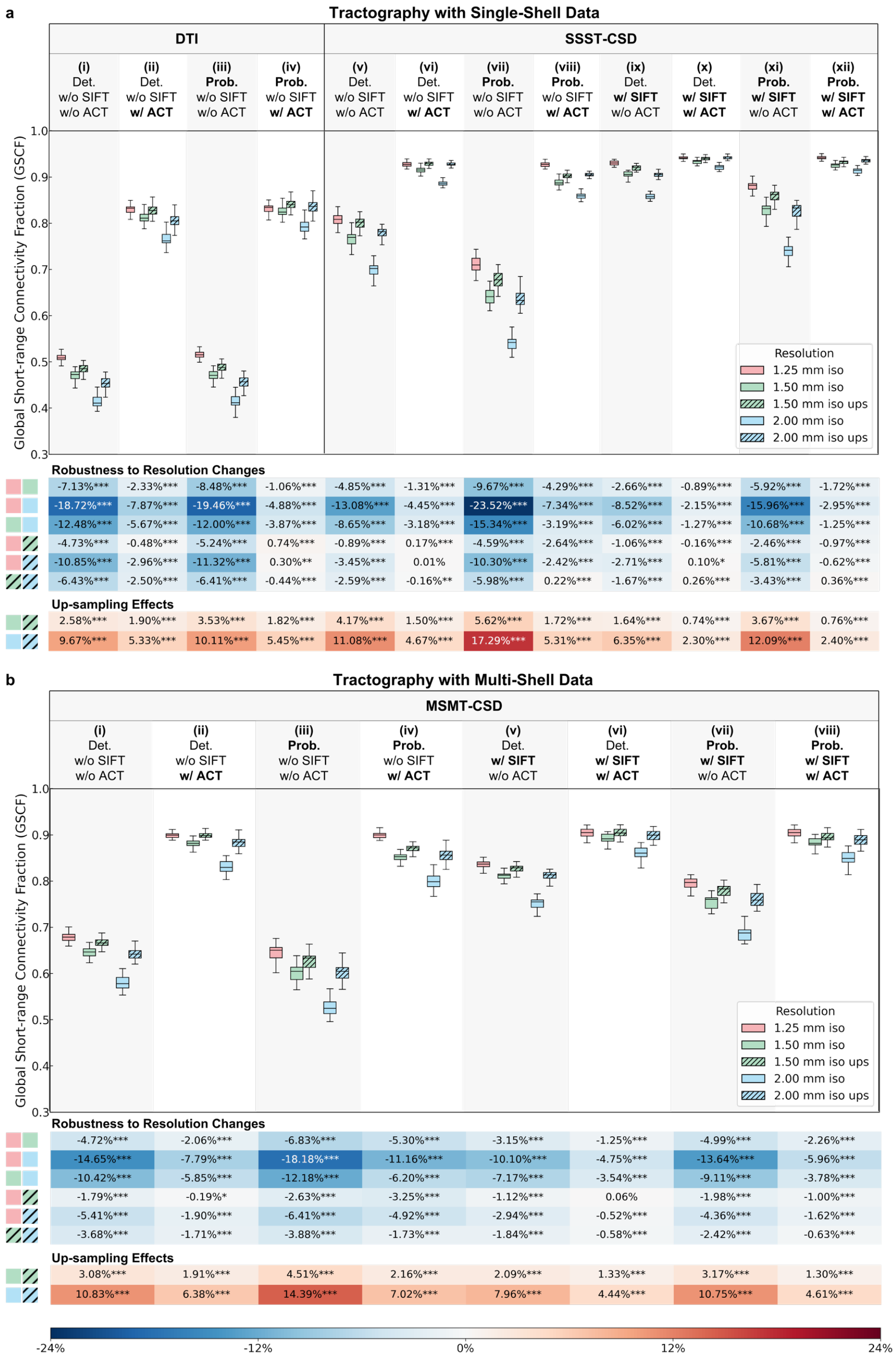

Figure S15. Retrospectively down-sampled data Variation 3 GSCF. Box plots for GSCF from different tractography methods using single-shell (a) and multi-shell (b) data at three native spatial resolutions (red: 1.25 mm iso., green: 1.5 mm iso., blue: 2 mm iso.) and two nominally high 1.25 mm iso. resolution up-sampled from 1.5 mm and 2 mm iso. resolutions (hatched green: 1.5 mm up-sampled, hatched blue: 2 mm up-sampled) display the distribution (i.e., median, interquartile range, and range) of GSCF from 20 subjects in the upper panel. The tables show the relative GSCF differences at lower resolutions compared to higher resolutions (upper tables: robustness to resolution changes) and at up-sampled nominal 1.25 mm resolution compared to native resolutions (lower tables: up-sampling effects) (each row) for different tractography methods (each column), with asterisks denoting significance levels (*: $p < 0.05$, **: $p < 0.01$, ***: $p < 0.001$). The color of each table cell indicates the magnitude and direction of the GSCF difference with a shared color-bar at the bottom.

Table S9. Spatial pattern of Variation 3 RSCF difference in retrospectively down-sampled data.

a. Cortical region most frequently ranked Top-10 RSCF difference

DTI		SSST-CSD		MSMT-CSD	
**cortex**	**freq.**	**cortex**	**freq.**	**cortex**	**freq.**
Left temporal pole	4/4	Right pars opercularis	7/8	Right superior temporal	7/8
Right superior temporal	3/4	Left pars opercularis	6/8	Right pars opercularis	6/8
Right transverse temporal	2/4	Right temporal pole	4/8	Left pars opercularis	6/8
Right rostral anterior cingulate	2/4	Right rostral anterior cingulate	4/8	Right inferior temporal	6/8
Right temporal pole	2/4	Right inferior temporal	4/8	Right frontal pole	5/8
Right medial orbitofrontal	2/4	Right caudal middle frontal	4/8	Right pars orbitalis	4/8
Right pars opercularis	2/4	Right insula	4/8	Right middle temporal	4/8
Right entorhinal	2/4	Left insula	4/8	Right temporal pole	4/8
Right precentral	2/4	Left temporal pole	4/8	Right inferior parietal	4/8
Left entorhinal	2/4			Left temporal pole	4/8
Left superior temporal	2/4				
Right inferior parietal	2/4				

b. Bilaterally recurring regions in a.

DTI	SSST-CSD	MSMT-CSD
Superior temporal	Temporal pole	Temporal pole
Temporal pole	Pars opercularis	Pars opercularis
Entorhinal	Insula	

Color key: red = temporal lobe; blue = parietal lobe; green = frontal lobe; orange = occipital lobe; gray = others (limbic lobe, insula, etc.)

Small-Worldness Calculation and Results

Small-Worldness (SW) quantifies the balance between local segregation and global integration of the structural connectome. A small-world brain network exhibits a high clustering coefficient (i.e. brain regions structurally connected to a particular region also tend to be interconnected with each other) and a short characteristic path length (i.e. strong neural pathways can generally be found between any two brain regions). SW is calculated as the ratio of the brain network's small-world properties to those of a random network. SW > 1 indicates that the brain network demonstrates stronger small-world properties compared to a random network. SW metrics were calculated using the GRETNA toolbox in MATLAB (Wang et al., 2015) described as follows.

The structural connectivity matrix W can be represented as a weighted, undirected graph $\mathrm{G} = (\mathrm{N}, \mathrm{E})$, where N is the set of nodes (cortical regions as defined by the Desikan-Killiany atlas) and E is the set of edges. An edge (i, j) exists between nodes i and j if their connectivity strength $\omega_{\mathrm{i,j}} > 0$. SW is calculated as the ratio of the normalized clustering coefficient (γ) to the normalized characteristic path length (λ) (Watts & Strogatz, 1998), separately for each hemispheric sub-graph $\mathrm{G_h}$ (where $\mathrm{h} \in \{\mathrm{left}, \mathrm{right}\}$):

$$\mathrm{SW}_h = \frac{\gamma_h}{\lambda_h}. \quad (1)$$

The normalized clustering coefficient $\gamma_\mathrm{h} = \mathrm{C_h}/\mathrm{C_{h,rand}}$ compares the average clustering coefficient $\mathrm{C_h}$ of the hemispheric network $\mathrm{G_h}$ to the average of value $\mathrm{C_{h,rand}}$ obtained from an ensemble (n = 100) of random networks. These random networks are generated by rewiring the original graph's edges while preserving the degree sequence (i.e., each node $\mathrm{k} \in \mathrm{N_h}$ maintains its original degree $\mathrm{d_k} = |\{\mathrm{i} \in \mathrm{N_h} \mid \omega_{\mathrm{ki}} > 0\}|$) (Maslov & Sneppen, 2002). The average clustering coefficient $\mathrm{C_h}$ is the mean of the local clustering coefficients $\mathrm{C_k}$ across all nodes $\mathrm{k} \in \mathrm{N_h}$. The local clustering coefficient $\mathrm{C_k}$ for a node k is calculated as (Onnela et al., 2005):

$$C_k = \frac{2}{d_k(d_k - 1)} \sum_{i,j \subset N_k} \left(\widehat{\omega}_{ki}\widehat{\omega}_{ij}\widehat{\omega}_{jk}\right)^{1/3} \tag{2}$$

where $N_k = \{i \in N_h \mid \omega_{ki} > 0\}$ is the set of neighbors of node k within the hemisphere h. The term $\widehat{\omega_{ij}} = \omega_{ij}/\max(\omega_h)$ represents edge weights normalized by the maximum weight in the hemispheric network G_h.

The normalized characteristic path length $\lambda_h = L_h/L_{h,\mathrm{rand}}$ compares the characteristic path length L_h of the network G_h to the average of value $L_{h,\mathrm{rand}}$ obtained from the same ensemble of degree-matched random networks. The characteristic path length L_h is the average shortest path length l_{ij} between all distinct node pairs $i, j \in N_h$. The shortest path length l_{ij} is defined as the minimum sum of inverse edge weights along any path $P_{ij} = (i = v_0, v_1, \dots, v_k = j)$ connecting nodes i and j:

$$l_{ij} = \min_{P_{ij}} \sum_{m=0}^{k-1} \frac{1}{\omega_{v_m v_{m+1}}} \tag{3}$$

where $\omega_{v_m v_{m+1}}$ is the weight of the edge between consecutive nodes v_m and v_{m+1} in the path P_{ij} (Dijkstra, 1959).

To mitigate the influence of potentially spurious weak connections (often false positives), a range of connection sparsity thresholds p (5% to 50% in 5% increments) was applied to each G_h to create $G_{h,p} = (N_h, E_{h,p})$, where $E_{h,p} \subseteq E_h$ such that $|E_{h,p}| = \lceil p \times |E_h| \rceil$ and for any $(u, v) \in E_{h,p}$ and $(x, y) \in E_h \setminus E_{h,p}$, $\omega_{uv} \geq \omega_{xy}$. For each $G_{h,p}$, $SW_{h,p}$ was calculated. This procedure generated a curve of $SW_{h,p}$ as a function of p. The area under this $SW_{h,p}$ versus p curve, integrated across the sparsity thresholds from 5% to 50%, was calculated. This integrated value was subsequently divided by 0.45 (corresponding to the span of a 5% to 50% sparsity range) to derive SW_h, a summary measure robust to threshold selection. The final SW value for the whole brain graph G was calculated as $(SW_{left} + SW_{right})/2$.

The statistical analysis across resolution was conducted using the same method with the statistics of GSCF.

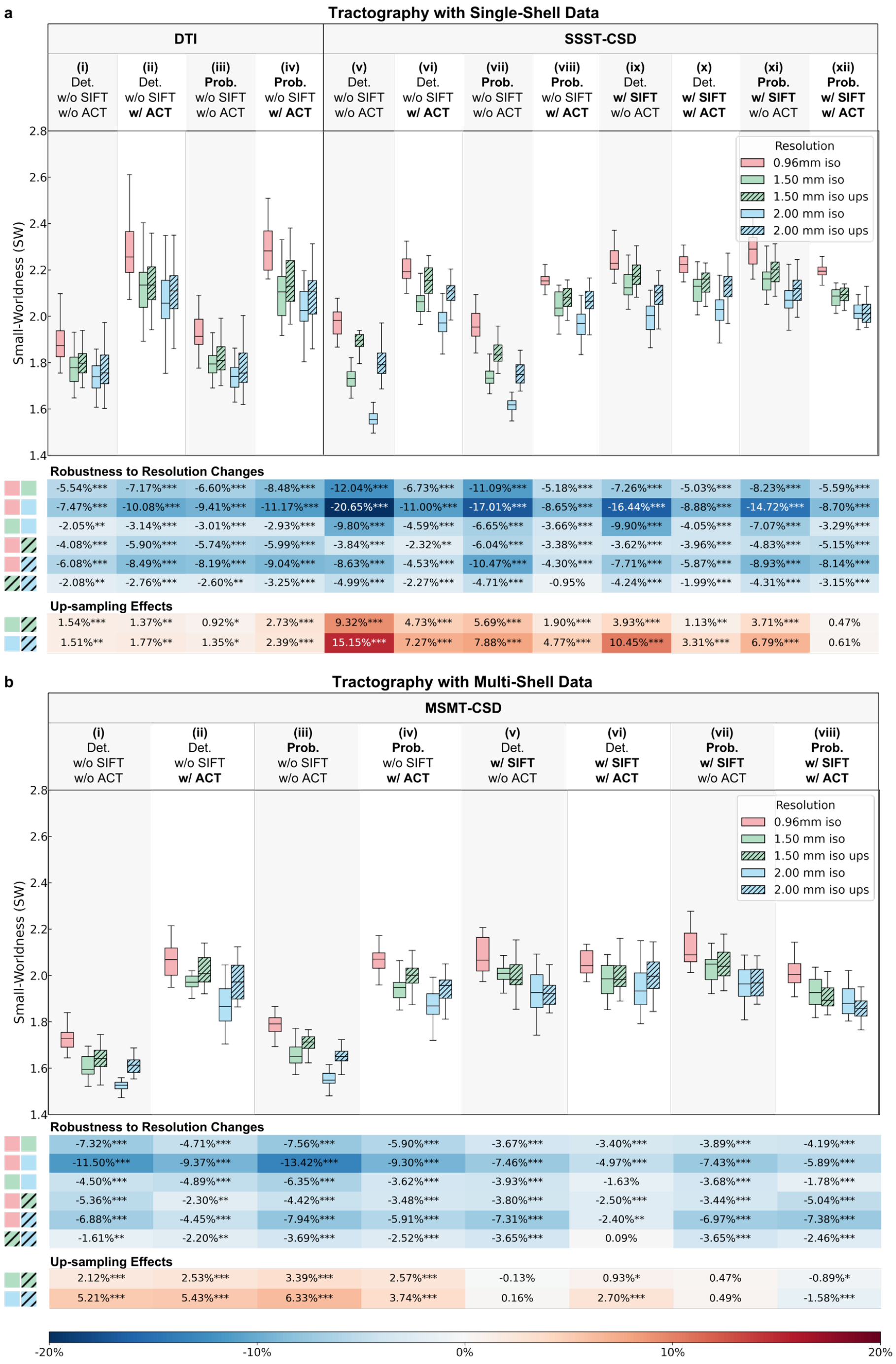

Figure S16 Prospectively acquired data SW. Box plots for SW from different tractography methods using single-shell (a) and multi-shell (b) data at three native spatial resolutions (red: 0.96 mm iso., green: 1.5 mm iso., blue: 2 mm iso.) and two nominally high 0.96 mm iso. resolution up-sampled from 1.5 mm and 2 mm iso. resolutions (hatched green: 1.5 mm up-sampled, hatched blue: 2 mm up-sampled) display the distribution (i.e., median, interquartile range, and range) of SW from 20 subjects in the upper panel. The tables show the relative SW differences at lower resolutions compared to higher resolutions (upper tables: robustness to resolution changes) and at up-sampled nominal 0.96 mm resolution compared to native resolutions (lower tables: up-sampling effects) (each row) for different tractography methods (each column), with asterisks denoting significance levels (*: $p < 0.05$, **: $p < 0.01$, ***: $p < 0.001$). The color of each table cell indicates the magnitude and direction of the SW difference with a shared color-bar at the bottom.

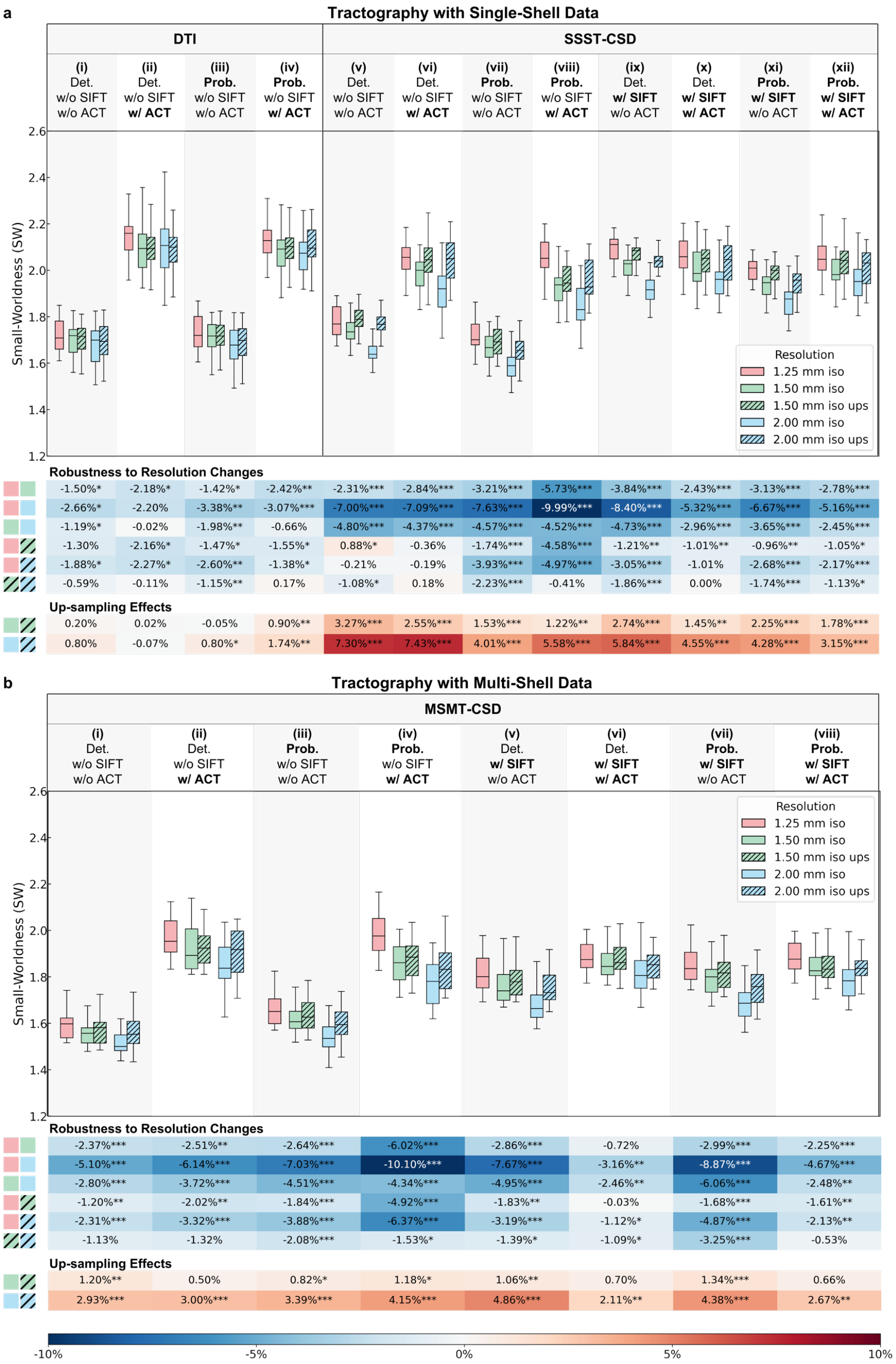

Figure S17. Retrospectively down-sampled data SW. Box plots for SW from different tractography methods using single-shell (a) and multi-shell (b) data at three native spatial resolutions (red: 1.25 mm iso., green: 1.5 mm iso., blue: 2 mm iso.) and two nominally high 1.25 mm iso. resolution up-sampled from 1.5 mm and 2 mm iso. resolutions (hatched green: 1.5 mm up-sampled, hatched blue: 2 mm up-sampled) display the distribution (i.e., median, interquartile range, and range) of SW from 20 subjects in the upper panel. The tables show the relative SW differences at lower resolutions compared to higher resolutions (upper tables: robustness to resolution changes) and at up-sampled nominal 1.25 mm resolution compared to native resolutions (lower tables: up-sampling effects) (each row) for different tractography methods (each column), with asterisks denoting significance levels (*: p < 0.05, **: p < 0.01, ***: p < 0.001). The color of each table cell indicates the magnitude and direction of the SW difference with a shared color-bar at the bottom.

Reference

Dijkstra, E. W. (1959). A note on two problems in connexion with graphs. *Numerische Mathematik*, *1*(1), 269–271. https://doi.org/10.1007/BF01386390

Maslov, S., & Sneppen, K. (2002). Specificity and Stability in Topology of Protein Networks. *Science*, *296*(5569), 910–913. https://doi.org/10.1126/science.1065103

Onnela, J. P., Saramäki, J., Kertész, J., & Kaski, K. (2005). Intensity and coherence of motifs in weighted complex networks. *Physical Review E - Statistical Physics, Plasmas, Fluids, and Related Interdisciplinary Topics*, *71*(6). https://doi.org/10.1103/PhysRevE.71.065103

Ricchi, M., Campani, G., Nagmutdinova, A., Bortolotti, V., Greco, D., Golini, C., Grist, J., Brizi, L., & Testa, C. (2025). Connectivity related to major brain functions in Alzheimer disease progression: Microstructural properties of the cingulum bundle and its subdivision using diffusion-weighted MRI. *European Radiology Experimental*, *9*(1), 32. https://doi.org/10.1186/s41747-025-00570-5

Saygi, T., Avyasov, R., Barut, O., Daglar, Z., Baran, O., Hasimoglu, O., Altinkaya, A., & Tanriover, N. (2023). Microsurgical anatomy of the isthmic cingulum: A new white matter crossroad and neurosurgical implications in the posteromedial interhemispheric approaches and the glioma invasion patterns. *Neurosurgical Review*, *46*(1), 82. https://doi.org/10.1007/s10143-023-01982-w

Schilling, K. G., Archer, D., Rheault, F., Lyu, I., Huo, Y., Cai, L. Y., Bunge, S. A., Weiner, K. S., Gore, J. C., Anderson, A. W., & Landman, B. A. (2023). Superficial white matter across development, young adulthood, and aging: Volume, thickness, and relationship with cortical features. *Brain Structure and Function*, *228*(3–4), 1019–1031. https://doi.org/10.1007/s00429-023-02642-x

Wang, J., Wang, X., Xia, M., Liao, X., Evans, A., & He, Y. (2015). GRETNA: A graph theoretical network analysis toolbox for imaging connectomics. *Frontiers in Human Neuroscience*, *9*, 386. https://doi.org/10.3389/fnhum.2015.00386

Watts, D. J., & Strogatz, S. H. (1998). Collective dynamics of “small-world” networks. *Nature*, *393*(6684), 440–442. https://doi.org/10.1038/30918

Yh, L., V, D., Ae, M., Im, Y., O, T., Rg, B., Ar, C., J, H., Rd, F., Sj, K., Jt, Y., C, T., & Me, S. (2021). Anatomy and White Matter Connections of the Parahippocampal Gyrus. *PubMed*. https://pubmed.ncbi.nlm.nih.gov/33412321/
